# A meta-analysis of genome-wide association studies for average daily gain and lean meat percentage in two Duroc pig populations

**DOI:** 10.1186/s12864-020-07288-1

**Published:** 2021-01-06

**Authors:** Shenping Zhou, Rongrong Ding, Fanming Meng, Xingwang Wang, Zhanwei Zhuang, Jianping Quan, Qian Geng, Jie Wu, Enqin Zheng, Zhenfang Wu, Jianhui Yang, Jie Yang

**Affiliations:** 1 College of Animal Science and National Engineering Research Center for Breeding Swine Industry, South China Agricultural University, Guangzhou, 510642 Guangdong People’s Republic of China; 2 State Key Laboratory of Livestock and Poultry Breeding / Guangdong Key Laboratory of Animal Breeding and Nutrition, Institute of Animal Science, Guangdong Academy of Agricultural Sciences, Guangzhou, 510640 Guangdong People’s Republic of China; 3 YueYang Vocational Technical College, Yueyang, 414000 Hunan People’s Republic of China

**Keywords:** Pigs, Lean meat percentage, Average daily gain, GWAS, Meta-analysis

## Abstract

**Background:**

Average daily gain (ADG) and lean meat percentage (LMP) are the main production performance indicators of pigs. Nevertheless, the genetic architecture of ADG and LMP is still elusive. Here, we conducted genome-wide association studies (GWAS) and meta-analysis for ADG and LMP in 3770 American and 2090 Canadian Duroc pigs.

**Results:**

In the American Duroc pigs, one novel pleiotropic quantitative trait locus (QTL) on *Sus scrofa* chromosome 1 (SSC1) was identified to be associated with ADG and LMP, which spans 2.53 Mb (from 159.66 to 162.19 Mb). In the Canadian Duroc pigs, two novel QTLs on SSC1 were detected for LMP, which were situated in 3.86 Mb (from 157.99 to 161.85 Mb) and 555 kb (from 37.63 to 38.19 Mb) regions. The meta-analysis identified ten and 20 additional SNPs for ADG and LMP, respectively. Finally, four genes (*PHLPP1*, *STC1*, *DYRK1B*, and *PIK3C2A*) were detected to be associated with ADG and/or LMP. Further bioinformatics analysis showed that the candidate genes for ADG are mainly involved in bone growth and development, whereas the candidate genes for LMP mainly participated in adipose tissue and muscle tissue growth and development.

**Conclusions:**

We performed GWAS and meta-analysis for ADG and LMP based on a large sample size consisting of two Duroc pig populations. One pleiotropic QTL that shared a 2.19 Mb haplotype block from 159.66 to 161.85 Mb on SSC1 was found to affect ADG and LMP in the two Duroc pig populations. Furthermore, the combination of single-population and meta-analysis of GWAS improved the efficiency of detecting additional SNPs for the analyzed traits. Our results provide new insights into the genetic architecture of ADG and LMP traits in pigs. Moreover, some significant SNPs associated with ADG and/or LMP in this study may be useful for marker-assisted selection in pig breeding.

## Background

Pork accounts for more than one-third of human meat consumption (http://www.fao.org/ag/againfo/themes/en/meat/background.html). Average daily gain (ADG) and lean meat percentage (LMP) are considered as growth and carcass traits of pigs and are important indicators of pig production performance, which directly affect the profit of the farm. For decades, breeders have improved ADG and LMP primarily through conventional breeding. However, ADG and LMP are complex quantitative traits regulated by multiple genes and improving these two traits through conventional breeding is time-consuming and expensive. Through molecular breeding, perhaps this technical bottleneck is solved. The rapid development of molecular markers and completion of pig genome sequence lay the foundation for molecular breeding in pigs [1, 2]. To date, quantitative trait locus (QTL) linkage analysis (termed QTL mapping) and genome-wide association analysis (GWAS) are two popular methods that are used to dissect the genetic architecture of complex traits in livestock. Numerous examples of successful QTL identification are available. Considering pigs as an example, 1916 and 16,147 QTLs are associated with growth traits and meat and carcass traits, including 692 and 172 QTLs associated with ADG and LMP in the pig QTL database (https://www.animalgenome.org/cgi-bin/QTLdb/SS/index, April 23, 2020) [3], respectively. However, poor resolution in QTL mapping experiments and complicated genetic architecture of many QTLs result in an unavoidable challenge for identifying causative mutations [4].

GWAS is considered as a powerful approach for detecting genetic factors related to phenotypes [5, 6]. With the development of high-density single nucleotide polymorphism (SNP) arrays and the reduction of high-density SNP analysis costs, GWAS has been widely used in domestic animals [4]. Previous GWAS for ADG and LMP traits usually used a limited number of animals (sample size < 1000) in pigs [7–10]. In general, with increasing the sample size, the power of GWAS to detect SNPs that are associated with the phenotype increases and the false-positive findings are reduced. However, due to the high cost of genotyping, most of the GWASs for economic importantly traits in livestock animals are performed based on the single-population of limited sample size, which consequently leads to insufficient detection power of association analysis. Meta-analysis is an effective method for solving the problem of insufficient sample size in GWAS. Meta-analysis can expand the sample size by combining multiple independent study data, thus increasing the power and reducing false-positive findings [11].

Here, we conducted GWASs and a meta-analysis for ADG and LMP in 3770 American and 2090 Canadian Duroc pigs. Our experimental population included a large sample size and comprised different Duroc pig populations to help detect novel QTLs and candidate genes for the analyzed traits.

## Results

### Phenotype and heritability statistics

As presented in Table 1, the genomic heritability of ADG and LMP traits ranged from 0.26 to 0.36. This study showed a positive correlation between ADG and LMP in American and Canadian Duroc pigs. In the American Duroc pig population, the phenotypic and genetic correlation coefficients reached 0.17 and 0.34 for ADG and LMP, respectively. In the Canadian Duroc pig population, the phenotypic and genetic correlation coefficients amounted to 0.09 and 0.20 for ADG and LMP, respectively (Table 1).

**Table 1:** Phenotype and heritability statistics for ADG and LMP in two Duroc populations

Population^1^	Traits^2^	N^3^	Mean (SD)^4^	C.V. (%)^5^	h^2^ (SE) ^6^	Phenotypic correlations^7^	Genetic correlations^8^
AD	ADG	3770	619.43 ± 32.70 (g)	5.28	0.26 ± 0.02	0.17	0.34 ± 0.06
	LMP	3769	62.24 ± 1.00 (%)	1.61	0.30 ± 0.02		
CD	ADG	2090	613.75 ± 43.26 (g)	7.05	0.28 ± 0.03	0.09	0.20 ± 0.07
	LMP	2082	61.02 ± 1.52 (%)	2.50	0.36 ± 0.03		

^1^American Duroc pig population (AD), Canadian Duroc pig population (CD). ^2^Average daily gain (ADG), lean meat percentage (LMP). ^3^Number (N). ^4^Mean (standard deviation). ^5^Coefficient of variation (C.V.). ^6^Heritability (standard error). ^7, 8^Phenotypic and genetic correlations (standard deviation) of ADG and LMP trait values, all of the phenotypic correlation coefficients are significant with *P* < 0.05

### Single-population GWAS results

**Fig. 1:**
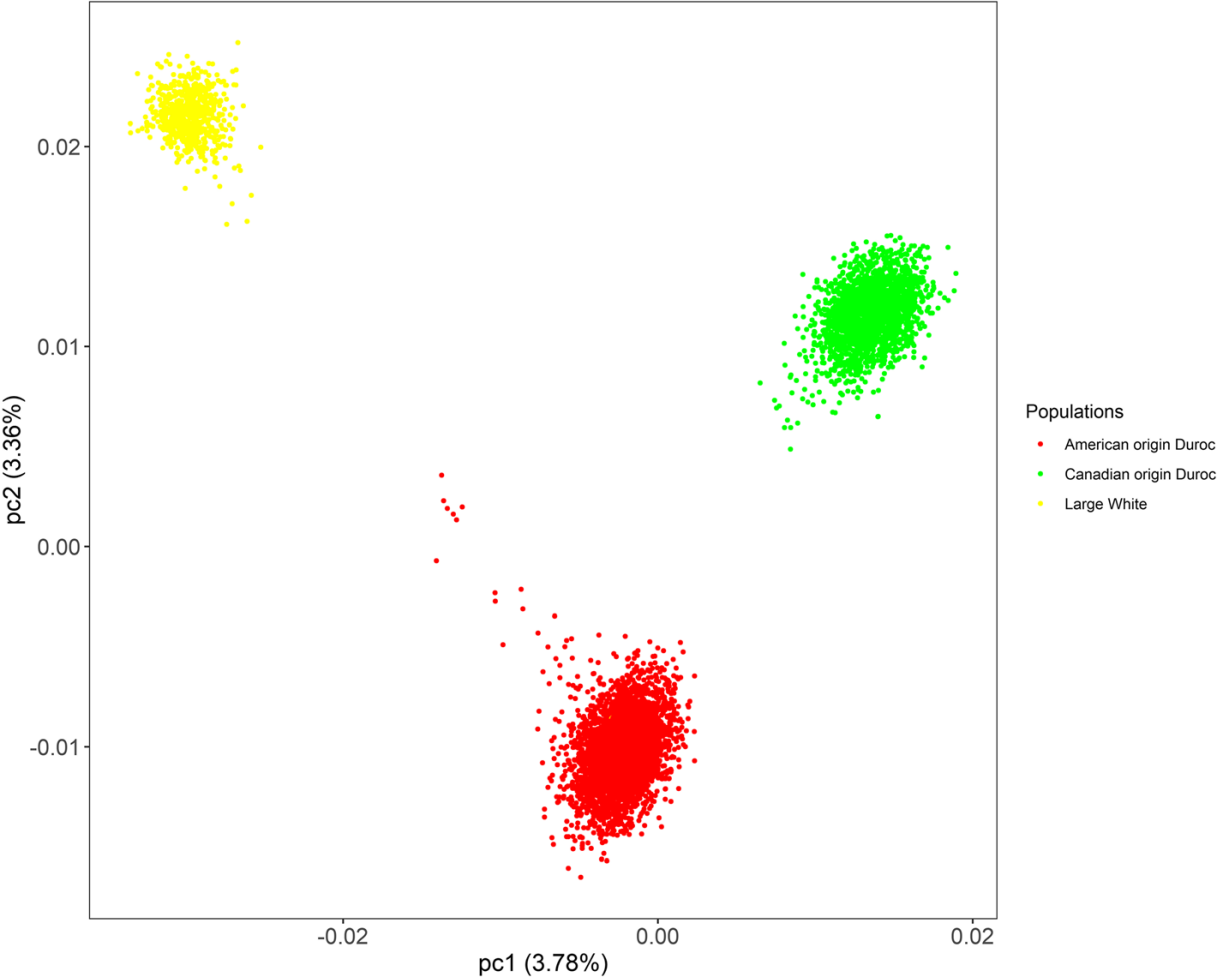
PCA plot of population structure showing the top two principle components. pc1: principle component 1; pc2: principle component 2. The red dot represents the American origin Duroc pigs, the green dot represents the Canadian origin Duroc pigs, and the yellow dot represents the Large White

Given that the experimental animals consisted of the two Duroc pig populations in this study, principal component analysis (PCA) was conducted to identify the potential population stratification. The PCA plot was shown in our previous paper [12]. Moreover, we added the 660 Large White pig population to compare further the differences in population structure of these two Duroc populations, and PCA was performed for the Large White and the two Duroc pig populations. As presented in Fig. 1, the PCA plot showed that the American and Canadian Duroc pigs did not coincide, indicating that these two Duroc pig populations have different genetic backgrounds. The quantile-quantile (Q–Q) plots showed that the ADG and LMP data for the two Duroc populations lack an overall systematic bias. The genomic inflation factor (λ) of GWAS ranged from 0.926 to 1.020, indicating that the systematic inflation of test statistics was not observed for the GWAS of both populations (Additional file 1: Figure S1).

**Fig. 2:**
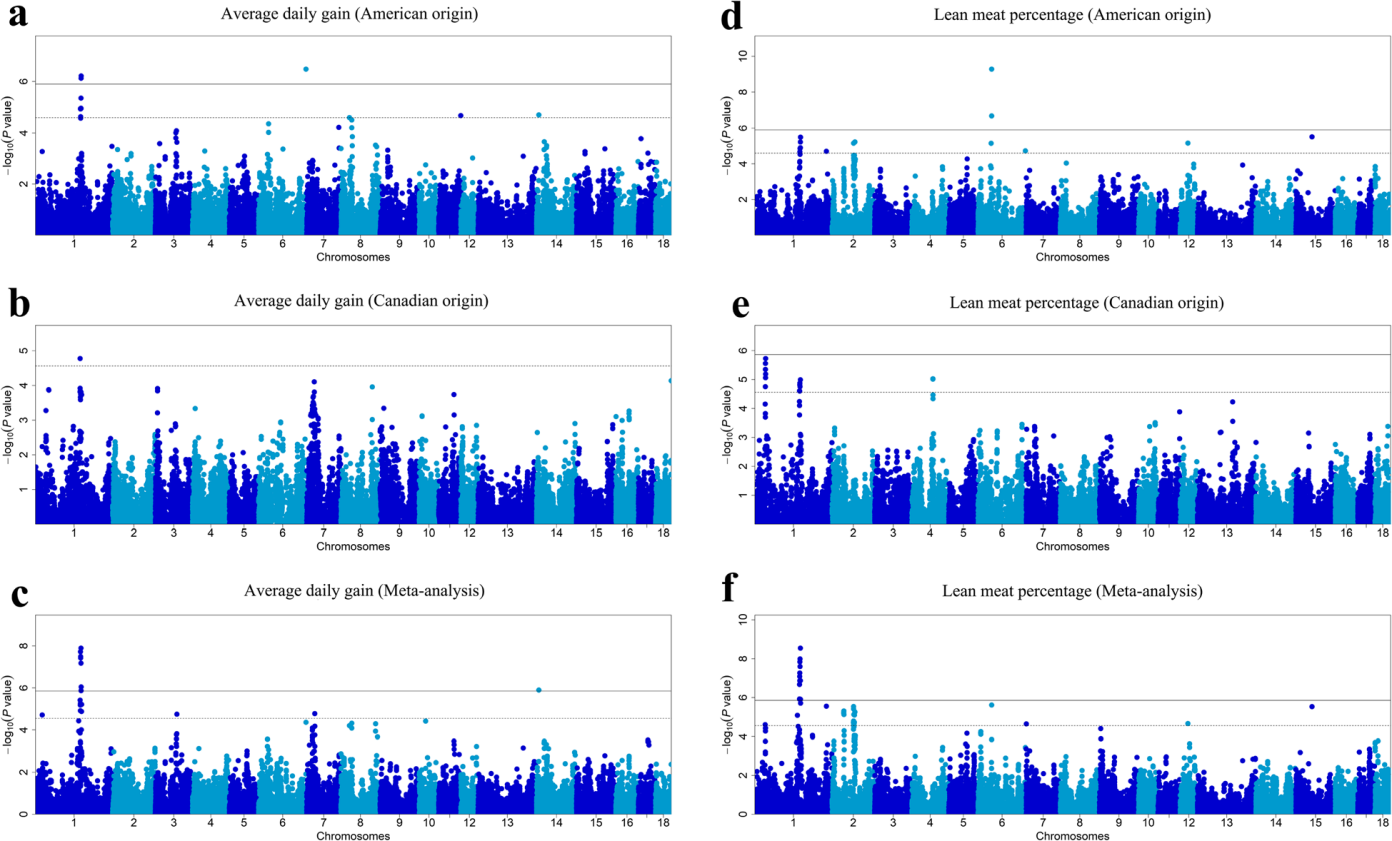
Manhattan plots of GWAS and meta-analysis for ADG and LMP in the two Duroc pig populations. In the Manhattan plots, the solid and dashed lines represent the 5% genome-wide and chromosome-wide (suggestive) Bonferroni-corrected thresholds, respectively. Manhattan plot for **a** Average daily gain (American origin), **b** Average daily gain (Canadian origin), **c** Average daily gain (Meta-analysis), **d** Lean meat percentage (American origin), **e** Lean meat percentage (Canadian origin), **f** Lean meat percentage (Meta-analysis)

After genotype quality control (QC), qualified SNPs were used for subsequent GWAS and meta-analysis (Additional file 2: Table S1). For the American Duroc pigs, seven suggestive (*P* < 2.82 × 10^− 5^) and three genome-wide (*P* < 1.41 × 10^− 6^) SNPs were identified to be associated with ADG; 13 suggestive and two genome-wide SNPs were detected to be associated with LMP (Table 2, Fig. 2). Interestingly, six SNPs on *Sus scrofa* chromosome 1 (SSC1) and one SNP on SSC6 were found to have pleiotropic effects on ADG and LMP (Table 2). For the Canadian Duroc pig population, one and 20 suggestive SNPs were identified to be associated with ADG and LMP, respectively (Table 2, Fig. 2). Notably, we observed three shared SNPs (MARC0013872, ASGA0004988, and ALGA0006623) by the American and Canadian Duroc pigs, and the SNPs may have pleiotropic effects on ADG and LMP in American Duroc pigs (Table 2).

**Table 2:** Significant SNPs and candidate genes for average daily gain and lean meat percentage in the single-population GWAS

Trait^1^ (population)	SNP^2^	SSC^3^	Location^4^ (bp)	EPV^5^ (%)	*P*-value^6^	Distance^7^ (bp)	Candidate gene
ADG (AD)	**H3GA0003149**	1	162,192,627	0.91	**6.07 × 10**^**− 7**^	within	*ALPK2*
	**WU_10.2_1_179575045**	1	161,987,727	0.92	**7.45 × 10**^**− 7**^	within	*ENSSSCG00000004911*
	**ALGA0006684**	1	161,853,405	0.93	4.49 × 10^− 6^	within	*ENSSSCG00000004911*
	**MARC0013872**	1	161,824,864	0.79	1.12 × 10^− 5^	within	*ENSSSCG00000004911*
	**ASGA0004988**	1	159,881,634	0.83	1.15 × 10^− 5^	within	*CDH20*
	**ALGA0006623**	1	160,347,188	0.76	2.31 × 10^− 5^	within	*ENSSSCG00000048538*
	**Affx-114,594,216**	6	168,268,278	0.65	**3.28 × 10**^**−7**^	13,851	*ENSSSCG00000039458*
	DIAS0000782	8	30,256,709	0.45	2.51 × 10^−5^	32,698	*TMEM156*
	WU_10.2_11_86301815	11	78,432,491	0.66	2.09 × 10^− 5^	within	*MCF2L*
	WU_10.2_14_8843751	14	7,988,327	0.24	1.97 × 10^−5^	−26,761	*STC1*
LMP (AD)	**WU_10.2_1_179575045**	1	161,987,727	0.47	3.22 × 10^− 6^	within	*ENSSSCG00000004911*
	**H3GA0003149**	1	162,192,627	0.47	3.24 × 10^− 6^	within	*ALPK2*
	**MARC0013872**	1	161,824,864	0.65	6.00 × 10^− 6^	within	*ENSSSCG00000004911*
	**ALGA0006684**	1	161,853,405	0.60	1.25 × 10^−5^	within	*ENSSSCG00000004911*
	**ALGA0006623**	1	160,347,188	0.59	1.38 × 10^−5^	within	*ENSSSCG00000048538*
	ALGA0123800	1	254,207,127	1.48	1.99 × 10^−5^	within	*RGS3*
	**ASGA0004988**	1	159,881,634	0.51	2.13 × 10^−5^	within	*CDH20*
	WU_10.2_2_82907810	2	81,306,158	1.44	5.91 × 10^−6^	− 3295	*SNCB*
	10,006,986	2	76,416,246	1.10	7.03 × 10^− 6^	within	*AMH*
	ASGA0096606	6	48,241,180	1.01	**5.17 × 10**^**−10**^	−31,313	*LGALS13*
	ASGA0091829	6	48,289,460	0.71	**2.07 × 10**^**−7**^	16,384	*DYRK1B*
	WU_10.2_6_41924003	6	46,461,817	0.87	7.05 × 10^−6^	within	*ZNF570*
	**Affx-114,594,216**	6	168,268,278	0.41	1.87 × 10^−5^	13,851	*ENSSSCG00000039458*
	ALGA0105911	12	27,109,190	1.23	6.92 × 10^−6^	− 8767	*WFIKKN2*
	WU_10.2_15_156432561	15	57,333,035	0.58	3.08 × 10^−6^	within	*ARHGEF4*
ADG (CD)	ASGA0004970	1	158,589,475	0.94	1.66 × 10^−5^	53,338	*PHLPP1*
LMP (CD)	ASGA0002401	1	38,161,769	2.75	1.87 × 10^−6^	within	*NKAIN2*
	MARC0034815	1	38,185,044	2.75	1.87 × 10^−6^	within	*NKAIN2*
	MARC0026342	1	38,189,919	2.75	1.87 × 10^−6^	within	*NKAIN2*
	H3GA0001475	1	37,366,714	2.18	2.78 × 10^−6^	− 135,329	*HEY2*
	DRGA0000591	1	37,381,311	2.12	4.48 × 10^−6^	−149,926	*HEY2*
	MARC0114211	1	37,401,594	2.12	4.48 × 10^−6^	− 170,209	*HEY2*
	DRGA0000604	1	38,067,414	2.09	6.49 × 10^−6^	within	*NKAIN2*
	ASGA0101182	1	37,746,276	2.06	8.68 × 10^−6^	within	*TPD52L1*
	MARC0013872	1	161,824,864	1.76	1.02 × 10^−5^	within	*ENSSSCG00000004911*
	ASGA0004988	1	159,881,634	1.74	1.33 × 10^− 5^	within	*CDH20*
	ALGA0006602	1	159,538,854	1.72	1.53 × 10^−5^	within	*RNF152*
	H3GA0003104	1	159,619,891	1.72	1.53 × 10^−5^	−17,910	*RNF152*
	H3GA0001466	1	37,024,102	1.18	1.76 × 10^−5^	−172	*HINT3*
	ALGA0006623	1	160,347,188	1.72	1.77 × 10^−5^	within	*ENSSSCG00000048538*
	WU_10.2_1_178188861	1	160,447,734	1.72	1.77 × 10^−5^	98,849	*ENSSSCG00000048538*
	INRA0004898	1	158,811,662	1.64	2.46 × 10^−5^	within	*PHLPP1*
	MARC0075909	1	159,238,083	1.66	2.51 × 10^−5^	within	*RELCH*
	ASGA0020293	4	74,693,408	1.38	9.37 × 10^−6^	within	*FAM110B*
	H3GA0013036	4	74,714,319	1.38	9.37 × 10^− 6^	within	*FAM110B*
	MARC0093868	4	74,774,997	1.38	9.66 × 10^−6^	−29,012	*FAM110B*

^1^*ADG* Average daily gain, *LMP* Lean meat percentage, *AD* American Duroc pig population, *CD* Canadian Duroc pig population. ^2^SNP ID in boldface represents the SNP had pleiotropic effects on ADG and LMP. ^3^*SSC Sus scrofa* chromosome. ^4^SNP positions in Ensembl. ^5^*EPV* Explained phenotypic variance. ^6^*P*-value in boldface: genome-wide significant; *P*-value not in boldface: suggestive significant. ^7^+/−: the SNP located upstream/downstream of the nearest gene

### Haplotype block analysis

**Fig. 3:**
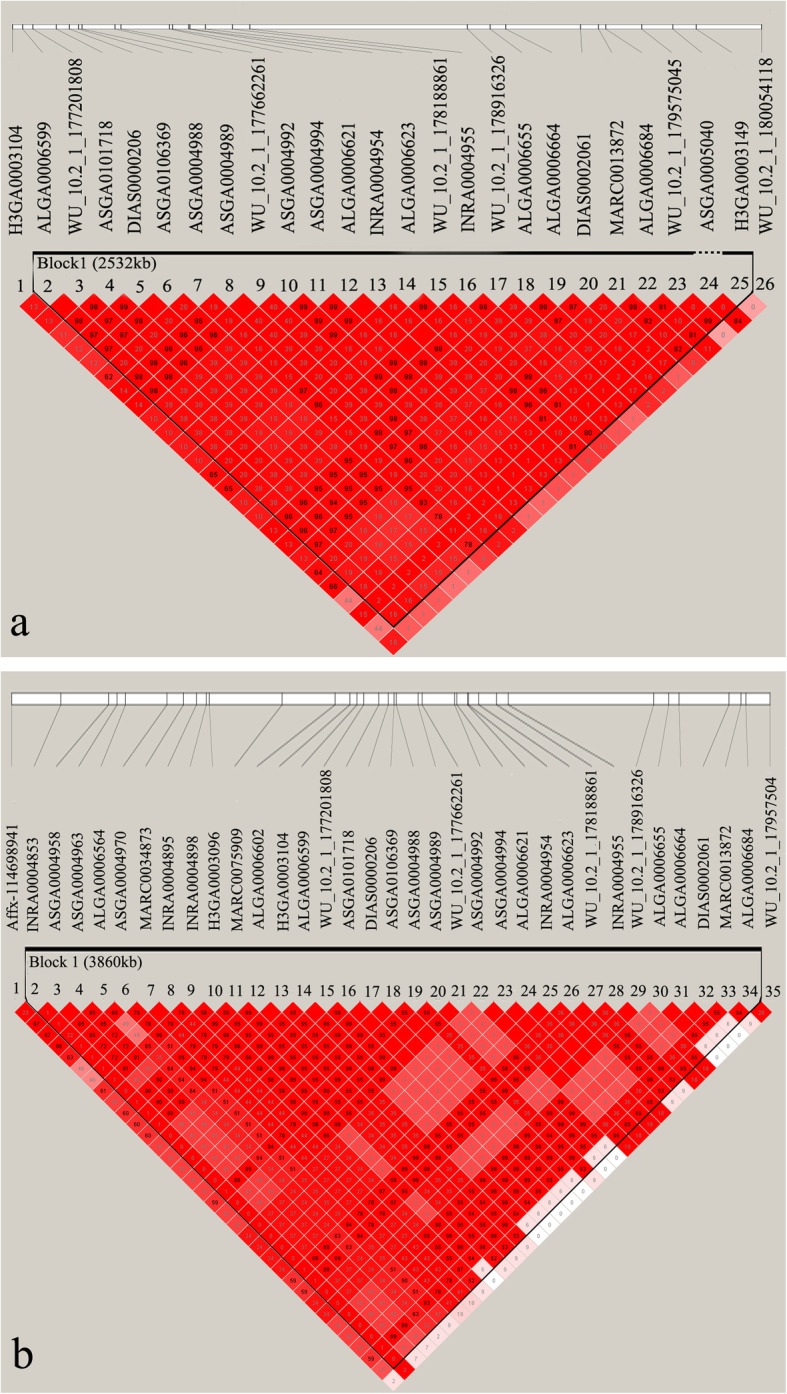
Haplotype blocks on SSC1. Haplotype blocks are marked with triangles. Values in boxes are the Linkage disequilibrium (r^2^) between the SNP pairs. The haplotype blocks are colored in accordance with the standard Haploview color scheme: LOD > 2 and D′ = 1, red; LOD < 2 and D′ < 1, white (LOD is the log of the likelihood odds ratio, a measure of confidence in the value of D′). Haplotype block for (**a**) average daily gain and lean meat percentage in American origin pigs, (**b**) lean meat percentage in Canadian origin pigs

In the American Duroc pigs, ten and 15 SNPs were identified to be associated with ADG and LMP, respectively (Table 2). Notably, six SNPs (ASGA0004988, ALGA0006623, MARC0013872, ALGA0006684, WU_10.2_1_179575045, and H3GA0003149) were associated with ADG and LMP, and these SNPs were situated in a haplotype block between 159.66 and 162.19 Mb (2.53 Mb) on SSC1 (Table 2 and Fig. 3a). In this QTL region, the primary SNP MARC0013872 had pleiotropic effects on ADG and LMP, and this SNP explained 0.79 and 0.65% of the phenotypic variance for ADG and LMP, respectively (Table 2). In the Canadian Duroc pigs, 20 SNPs were detected to be associated with LMP (Table 2). Among them, five SNPs (ASGA0101182, DRGA0000604, ASGA0002401, MARC0034815, and MARC0026342) were located in a haplotype block between 37.63 and 38.19 Mb (555 kb) on SSC1. The marker ASGA0002401 was the most significant SNP for this QTL region (Table 2 and Additional file 3: Figure S2). Moreover, eight identified SNPs (MARC0013872, ALGA0006623, ASGA0004988, WU_10.2_1_178188861, ALGA0006602, MARC0075909, H3GA0003104, and INRA0004898) lie in a haplotype block between 157.99 and 161.85 Mb (3.86 Mb) on SSC1, of which three SNPs were shared by the two Duroc pig populations. The marker MARC0013872 was the most significant SNP for this QTL region (Table 2 and Fig. 3b). The top markers ASGA0002401 and MARC0013872 for the above characterized haplotype blocks explained 2.75 and 1.76% of the phenotypic variance for LMP, respectively (Table 2).

**Fig. 4:**
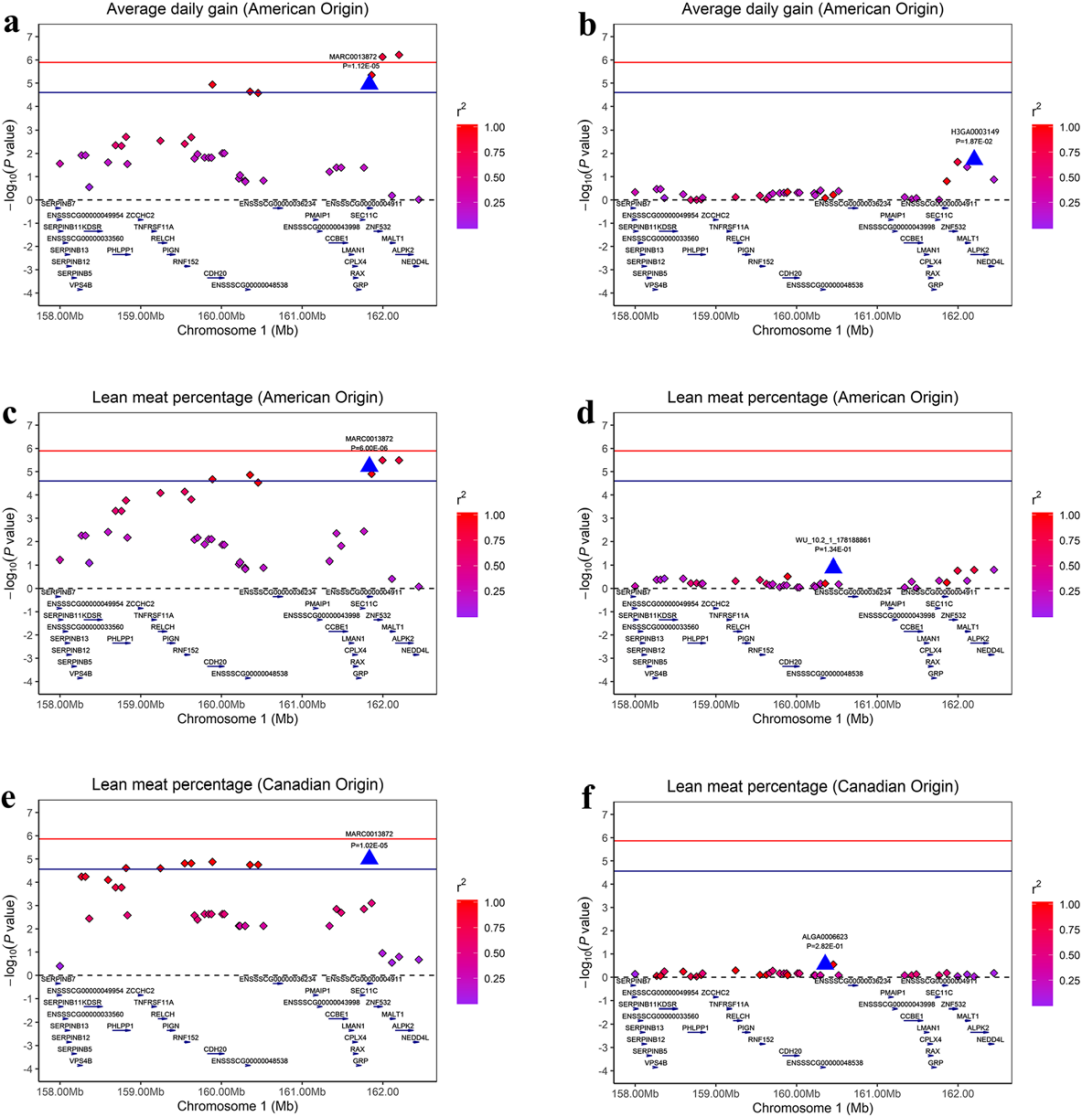
Regional association plots of the significant SNP (MARC0013872) associated with ADG and LMP at SSC1. In the plots, the red and blue represent the 5% genome-wide and chromosome-wide (suggestive) Bonferroni-corrected thresholds, respectively. The significant SNP are indicated by big blue triangles. SNPs are denoted by colored diamonds depending on the target SNP with which they were in strongest LD. The plots indicate the association results for ADG on American origin Duroc pigs (**a**) before and (**b**) after conditional analysis on MARC0013872. The plots indicate the association results for LMP on American origin Duroc pigs (**c**) before and (**d**) after conditional analysis on MARC0013872. The plots indicate the association results for LMP on Canadian origin Duroc pigs (**e**) before and (**f**) after conditional analysis on MARC0013872

To examine whether linkage disequilibrium (LD) led to the associations, we conducted conditional analyses for the primary two SNPs (MARC0013872 and ASGA0002401) that were fitted into the univariate linear mixed model as a covariate in GEMMA [13]. The results showed that many significant SNPs were in high LD status with the primary SNP MARC0013872, for which the *P*-values decreased below the minimum threshold line (Fig. 4). Similarly, the same pattern was also observed after conditional analysis for the SNP ASGA0002401 (Additional file 4: Figure S3).

### Meta-analysis of GWAS results

For ADG and LMP traits, this study pooled data from a GWAS conducted on American and Canadian Duroc pigs for meta-analysis. The meta-analysis detected nine suggestive and eight genome-wide SNPs associated with ADG, of which ten SNPs were undetected in both of the single-population GWAS (Additional file 5: Table S2 and Additional file 6: Figure S4a). Seven SNPs identified in the American Duroc pig were confirmed by meta-analysis. Furthermore, the meta-analysis identified 25 suggestive and 12 genome-wide SNPs to be associated with LMP, of which 20 SNPs were undetected in both of the single-population GWAS (Additional file 5: Table S2 and Additional file 6: Figure S4b). Nine and five SNPs respectively identified in the American and Canadian Duroc pigs were confirmed by meta-analysis, together with three common SNPs in the two Duroc pig populations (Additional file 5: Table S2 and Additional file 6: Figure S4b). Overall, for ADG and LMP traits, the meta-analysis confirmed 24 SNPs identified in the single-population GWAS, of which 20 SNPs had smaller *P*-values than in the single-population GWAS, including five and 15 SNPs associated with ADG and LMP, respectively (Table 2 and Additional file 5: Table S2). As shown in Additional file 6: Figure S4, using the meta-analysis, missing SNPs that were undetected by the single-population GWAS were retrieved and showed the advantage of meta-analysis that it can integrate results across populations to avoid the influence of population stratification.

### Comparison with previously mapped QTL in pigs

To evaluate whether QTLs associated with ADG and/or LMP traits in this study replicate any previously known QTLs, the pigQTLdb was searched on the basis of SNP and QTL locations.

For ADG, a total of 21 SNPs were identified, of which 18 SNPs are located in previously reported QTL regions of the ADG trait in pigs (Additional file 7: Table S3). The remaining 3 SNPs have not been included in any previously reported QTLs that are associated with ADG of pigs (Additional file 8: Table S4). For LMP, a total of 52 SNPs were detected, of which ten SNPs are located in previously reported QTL regions of the LMP in pigs (Additional file 7: Table S3). The remaining 42 SNPs have not been included in any previously reported QTLs that are associated with LMP of pigs (Additional file 8: Table S4).

### Candidate genes and functional analysis

A total of 39 functional genes that were within or near the identified significant SNPs were detected based on annotations of the *Sus scrofa* 11.1 genome assembly (Table 2 and Additional file 5: Table S2). Kyoto Encyclopedia of Genes and Genomes (KEGG) and Gene Ontology (GO) analyses were performed to highlight pathways and biological processes for ADG and LMP in pigs. For ADG, the KEGG pathways and GO terms are enriched for the candidate genes, including bone growth and development, calcium ion transport, etc. (Additional file 9: Table S5). For LMP, the KEGG pathways and GO terms are enriched for the candidate genes, including adipose tissue and muscle tissue growth and development, etc. (Additional file 10: Table S6).

## Discussion

### Single-population GWAS versus meta-analysis of GWAS

In this study, we conducted a combination strategy (included single-population GWAS and meta-analysis) for ADG and LMP in 3770 American and 2090 Canadian Duroc pigs. Because these two Duroc pig populations have different genetic backgrounds in this study, single-population GWASs were implemented. Meta-analysis can increase power, reduce false-positive findings, and even identify some new genetic loci, so it can solve the shortcomings of single-population GWASs [11, 14]. Meta-analysis has become a popular approach for the discovery of new genetic loci for common phenotypes and has been widely used in domestic animals [15, 16]. Thus, we conducted meta-analyses by combining the results of the two single-population GWASs in this study. To prove the superiority of this combination strategy, we conducted a mixed-population GWAS in these two Duroc pig populations. Through the Venn plot (Additional file 6: Figure S4), we found that there were many significant SNPs detected by the meta-analysis than the mixed-population GWAS in the two traits (ADG: 17 vs 12; LMP: 37 vs 22), and the proportion of significant SNPs detected by the single population GWAS verified by the meta-analysis is also higher than the mixed population GWAS (ADG: 7/17 vs 1/12; LMP: 17/37 vs 9/22), which implies that the meta-analysis may be more suitable. Moreover, many previous studies used single-population GWAS and meta-analysis to detect SNPs that were associated with phenotypes [17–19]. Thus, we used this combination strategy to analyze the genetic analysis of ADG and LMP traits in two Duroc populations.

Our results showed that three significant SNPs (MARC0013872, ASGA0004988, and ALGA0006623) were found simultaneously to be associated with ADG and LMP in both single-population GWASs. Many previous studies also detected a few or no shared SNPs in pigs of different breeds or populations belonging to the same breed. For instance, Jiang et al. [20] performed GWAS for backfat thickness and days to 100 kg in 2025 pigs from American and British Yorkshire pig populations, and no significant SNPs shared by these two populations were detected. Thu et al. [21] performed GWAS for conformation traits in three populations (Landrace, Yorkshire, and Duroc pigs) and found that an overlapping region exists on SSC7 in Yorkshire and Duroc. Furthermore, the American and Canadian Duroc pig populations have different genetic backgrounds in this study. Thus, we considered that these differences of the single-population GWAS results between American and Canadian Duroc pig populations might indicate genetic heterogeneity of ADG and LMP between the two Duroc pig populations. Moreover, these differences of GWAS results also showed that genetic backgrounds could substantially influence single-marker associations and further confirmed the complex genetic architecture of ADG and LMP in pigs. Additional researches are needed to explore the reason of existing large differences for ADG or LMP between different pig populations and further dissect the genetic basis of these traits in pigs.

Furthermore, the meta-analysis detected several SNPs that were undetected in the single-population GWAS, including ten significant SNPs for ADG and 20 significant SNPs for LMP. Besides, for both ADG and LMP, the meta-analysis results confirmed 24 SNPs that were identified in the single-population GWAS, of which 20 SNPs had smaller *P*-values than in the single-population GWAS. A similar trend was observed in previous studies. For instance, He et al. [22] conducted GWAS and meta-analysis for internal organs on 2650 pigs from five populations. The meta-analysis detected four additional QTLs for carcass weight and confirmed most of the significant SNPs in the single-population GWAS, of which 23.2% of the confirmed SNPs had smaller *P*-values than the single-population GWAS. Liu et al. [19] performed a meta-analysis of GWAS for meat quality traits in four pig populations, and seven novel QTLs were detected by meta-analysis. These findings showed that meta-analysis could expand the sample size to increase the power of GWAS by combining multiple independent studies, identified some additional SNPs and confirm a number of SNPs that were detected in single-population GWAS.

### Comparison of QTLs identified in this study with findings of previous studies

To further identify candidate regions associated with ADG and LMP traits, haplotype block analyses were conducted for chromosomal regions with multiple significant SNPs clustered around the top SNP. In this study, one QTL on SSC1 was detected to be associated with ADG and LMP in the American Duroc pigs, which spans approximately 2.53 Mb (from 159.66 to 162.19 Mb). In the Canadian Duroc pigs, one QTL also on SSC1 was identified to be associated with LMP, which was mapped to a 3.86 Mb interval (from 157.99 to 161.85 Mb). Interestingly, the two Duroc pig populations shared a 2.19 Mb haplotype block from 159.66 to 161.85 Mb. Furthermore, five SNPs, located in a 555 kb interval (from 37.63 to 38.19 Mb) on SSC1, were newly detected to be associated with LMP in the Canadian Duroc pigs. Notably, the three QTLs have not been included in any previously reported QTLs that are associated with LMP of pigs. However, given the large interval of these three QTLs, they still need to be further fine mapped, to be better applied to improve ADG and LMP in future pig breeding programs.

### Candidate genes

In this study, four genes related to ADG and/or LMP of pigs were selected as candidates. In the four candidate genes, one gene (Stanniocalcin 1 [*STC1*]) was associated with ADG, two genes (Phosphatidylinositol-4-Phosphate 3-Kinase Catalytic Subunit Type 2 Alpha [*PIK3C2A*], and Dual Specificity Tyrosine Phosphorylation Regulated Kinase 1B [*DYRK1B*]) were related to LMP, and one gene (PH Domain And Leucine Rich Repeat Protein Phosphatase 1 [*PHLPP1*]) affected ADG and LMP.

For ADG, one significant SNP (WU_10.2_14_8843751) on SSC14 is located nearest the *STC1* gene, and this gene was reported to affect growth, muscle mass, and bone size in transgenic mice [23, 24]. Therefore, the *STC1* gene may be a crucial factor for body growth and development, and this gene should be considered a strong candidate gene for ADG. For LMP, one significant SNP DIAS0000957 on SSC2 is located within the gene *PIK3C2A*. The *PIK3C2A* protein inactivation causes relative muscle mass loss and increased adipose tissue in mice [25]. Moreover, the *DYRK1B* gene is near SNP ASGA0091829, and this SNP was detected to be associated with LMP in this study. Keramati et al. [26] reported that non-mutant protein encoded via *DYRK1B* restrains sonic hedgehog and Wnt signaling pathways to promote adipogenesis. As is known, fat and muscle content are the main factors affecting LMP, so these two genes should be regarded as strong candidate genes for LMP in pigs. For ADG and LMP, four significant SNPs on SSC1 are located within or nearest the *PHLPP1* gene, of which two SNPs (ASGA0004970 and INRA0004898) were detected to be associated with these two traits. Notably, these four SNPs were mapped to a haplotype block spanning 3.86 Mb (from 157.99 to 161.85 Mb) for LMP in Canadian pigs. *PHLPP1* gene was reported to affect Akt signaling, and Akt signaling regulates chondrocyte proliferation, suggesting the *PHLPP1* gene may be involved in bone development [27, 28]. In addition, Caricilli et al. [29] found that weight loss in obese mice was related to reduced *PHLPP1* protein expression in the hypothalamus. These results indicated that the *PHLPP1* gene may play an important role in ADG and LMP, and should be considered a strong candidate gene for the two traits.

Functional annotation revealed several terms and pathways that related to the biological process of ADG and LMP (Additional file 9: Table S5 and Additional file 10: Table S6). For ADG, the significant terms are mainly involved in bone growth and development, and calcium ion transport. Calcium phosphate is a component of bones, and bones can serve as the primary storage of calcium and phosphate ions required for various metabolic functions [30]. Given that the ADG is considered as growth traits of pigs, it is conceivable that the candidate genes for ADG participated in bone growth and development. For LMP, the significant terms and pathways were mainly participated in adipose tissue and muscle tissue growth and development. Knecht and Duziński [31] demonstrated that lean meat content is positively correlated with muscle content and negatively correlated with the amount of subcutaneous fat with skin in pigs. These results indicated that muscle and adipose content affect the LMP trait of pigs.

## Conclusions

We performed GWAS and meta-analysis for ADG and LMP in 5860 Duroc pigs from there are two populations of the same breed. A total of 59 SNPs were identified in this study, and the meta-analysis identified 13 and 18 additional SNPs to be associated with ADG and LMP, respectively. Interestingly, one pleiotropic QTL that shared a 2.19 Mb haplotype block from 159.66 to 161.85 Mb on SSC1 was identified to affect ADG and LMP in the two Duroc pig populations. Furthermore, five SNPs, located in a 555 kb QTL (from 37.63 to 38.19 Mb) on SSC1, were newly detected to be associated with LMP in the Canadian Duroc pigs. Fine mapping is needed to further narrow the interval of these two QTLs for ADG and/or LMP in pigs. Our results provide new insights into the genetic architecture of ADG and LMP traits in pigs. Moreover, some significant SNPs associated with ADG and/or LMP in this study may be useful for marker-assisted selection in pig breeding.

## Methods

### Animals and phenotype

Between 2013 and 2017, 3770 American Duroc and 2090 Canadian Duroc pigs were raised in two breeding farms in Guangdong Wen’s Foodstuffs Co., Ltd. (Guangdong, China). When these 5860 Duroc pigs reached the body weight of 30 ± 5 kg, the pigs were transferred to the test station. The pigs were freely fed, provided with drinking water, and measured with a final weight of 100 ± 5 kg in the test station. Phenotypic records included ADG and LMP. When their body weight approximately reached 100 kg, the ADG and age to 100 kg (AGE) traits were measured, and then adjusted to 100 kg. The corrected 100 kg AGE was calculated by the following formula [32]:

$$ AGE\ (day)= Measured\  age-\left[\left( Measured\ body\ weight-100\  kg\right)/ CF\right] $$

where correction factors (CF) are different for male and female, and the CF was used in the following formula:

$$ Male: CF= Measured\ body\ weight/ Measured\  age\ast 1.826 $$

$$ Female: CF= Measured\ body\ weight/ Measured\  age\ast 1.715 $$

the corrected 100 kg ADG was calculated by the following formula [32]:

$$ ADG\ \left( kg/ day\right)=100 kg/ AGE $$

In addition, when their body weight reached 100 ± 5 kg, the backfat thickness (BF) and loin muscle depth (LMD) of the pigs were measured by an Aloka 500 V SSD B ultrasound (Corometrics Medical Systems, USA) between the 10th and 11th rib of the pig. The phenotypic values of LMP were calculated based on the BF and LMD as following described [33]:

$$ LMP\ \left(\%\right)=61.21920-0.77665\ast BF+0.15239\ast LMD $$

where *LMP* denotes the corrected 100 kg LMP; *BF* represents the corrected 100 kg BF; *LMD* signifies the corrected 100 kg LMD. The corrected 100 kg BF and LMD were obtained based on the Canadian Centre for Swine Improvement (http://www.ccsi.ca/Reports/Reports_2007/Update_of_weight_adjustment_factors_for_fat_and_lean_depth.pdf) as the following formula:

$$ BF\ (mm)= Measured\  BF\ast \frac{A}{A+B\ast \left( Measured\ body\ weight-100\  kg\right)} $$

where *A* and *B* are different for male and female, and the values represented by *A* and *B* are as follows:

$$ male:A=13.47;B=0.1115 $$

6 $$ female:A=15.65;B=0.156 $$

the corrected 100 kg LMD was calculated by the following formula:

$$ LMD\ (mm)= Measured\  LMD\ast \frac{a}{a+b\ast \left( Measured\ body\ weight-100\  kg\right)} $$

where *a* and *b* vary by the gender of the individual, and the values specified by *a* and *b* are as follows:

$$ male:a=50.52;b=0.228 $$

$$ female:a=52.01;b=0.228 $$

### Genotype data acquisition and quality control

Collected ear tissues were used to extract genomic DNA by applying standard protocols. In this study, all animals were still raised until elimination after phenotypic measurement and ear tissue collection. These animals were not anesthetized during the ear tissue collection. Genotyping was conducted as described by Ding et al. [34]. DNA quality was measured by electrophoresis and a light absorption ratio (A260/280). All DNA samples were diluted to a concentration of 50 ng/μL. After DNA QC, 5860 Duroc and pigs were genotyped with the Geneseek Porcine 50 K SNP chip (Neogen, Lincoln, NE, United States). The genotype QC was conducted by PLINK v 1.9 [35]. The following criteria were used to filter SNPs prior to conducting association analysis: animal call rates < 95%, SNPs with call rates of < 90%, minorallele frequency < 1%, and *P-*value < 10^− 6^ for the Hardy-Weinberg equilibrium test were excluded. This study excluded the SNPs located on the sex chromosomes and unmapped regions. In addition, the two Duroc pig populations follow the same QC criteria. In the meta-analysis of GWAS, a common set of SNPs that passed QC across the two populations were later used.

### Assessing the power of GWAS

In this study, the sample size calculation was based on the following two formulas, in which the correlation (r) between the marker and the trait was calculated by the following formula [36]:

$$ r\left(t,m\right)=r\left(m,q\right)\ast r\left(q,g\right)\ast r\left(g,t\right) $$

where *m* denotes the marker genotype; *q* represents the QTL genotype; *g* is the genetic value; *t* is the phenotypic value. *r*^*2*^*(m,q)* specifies the conventional r^2^ measure of linkage disequilibrium; *r*^*2*^*(q,g)* stands for the proportion of genetic variance explained by the QTL; *r*^*2*^*(g,t)* refers to the heritability of the trait. In this study, we assumed that *r*^*2*^*(m,q)* = 0.35, *r*^*2*^*(q,g)* = 0.04. *r*^*2*^*(g,t)* is the heritability value estimated by GCTA software.

The number of animals (*N*) required for detecting a QTL was calculated by the following formula [36, 37]:

$$ N={\left(\frac{1-{r}^2\left(t,m\right)}{r\left(t,m\right)\left(1/{Z}_{\left(1-\alpha \right)}\right)}\right)}^2 $$

where *Z* denotes the normal score; *α* represents the Bonferroni-corrected type I error rate for *K* independent tests, in which *K* is the number of effective SNPs.

According to the formulas, the number of animals required for ADG and LMP are 4490 and 3889, respectively. Herein, we conducted GWASs for ADG and LMP in 3770 American and 2090 Canadian Duroc pigs. To avoid the insufficient detection power of single-population-based GWAS and improve the efficiency of identifying QTL, we conducted meta-analyses in 5860 pigs from the two Duroc pig populations.

### Population structure analysis

Population stratification vastly affects GWAS reliability, so software R and GCTA were used to evaluate the population structure of two Duroc populations [38, 39]. The Q–Q plot is a commonly used tool for scanning population stratification in GWAS studies [40]. In this study, the Q–Q plot was constructed by R v3.6.1 software. Given that the experimental animals originated from two groups in this study, PCA was used to evaluate the similarity of the genetic background between American and Canadian Duroc pigs. PCA was generated by software GCTA v1.92.4beta.

### Single-population GWAS

GEMMA software was applied to a univariate linear mixed model to execute GWAS, and the single-population analysis of the two Duroc pig populations used the same univariate linear mixed model [13]. Before GWAS, the genomic relatedness matrix (GRM) between individuals was estimated by GEMMA. The matrix form was used in the following statistical model:

$$ y= W\alpha + X\beta +u+\varepsilon $$

where *y* refers to a vector of phenotypic values for all animals; *W* denotes the incidence matrices of covariates (fixed effects), including sex, live weight, and the top three eigenvectors of PCA; *α* represents the vector of corresponding coefficients with the intercept; *X* corresponds to the vector of marker genotypes; *β* specifies the corresponding effect size of the marker; *u* stands for the vector of random effects with *u* ~ MVN_n_ (0, λ τ^− 1^K); *ε* is the vector of random residuals with *ε* ~ MVN_n_ (0, τ^− 1^In); *λ* signifies the ratio between two variance components; τ^−1^ is the variance of the residual errors; *K* serves as GRM; *I* is an n × n identity matrix; MVN_n_ denotes the n-dimensional multivariate normal distribution.

Moreover, to prove the superiority of the combination strategy (included single-population GWAS and meta-analysis), we conducted GWAS for ADG and LMP traits in a mixed-population (included these two Duroc pig populations) and added population as a covariate into the univariate linear mixed model.

### Meta-analysis of GWAS

Meta-analysis was conducted by combining single-population GWAS analysis using METAL software [41]. In this study, METAL combined the results of the two single-population GWASs by calculating the pooled inverse-variance-weighted *β*-coefficients, standard errors, and Z-scores, and the formulas were as follows:

$$ {w}_i=1/{SE}_I^2 $$

$$ se=\sqrt{1/\sum \limits_i{w}_i} $$

$$ \beta =\sum \limits_i{\beta}_i{w}_i/\sum \limits_i{w}_i $$

$$ Z=\beta / SE $$

where *β*_*i*_ is the *β*-coefficients for study i; *SE* corresponds to the standard errors for study i.

In the single-population GWAS and meta-analysis, the genome-wide significant (0.05/N) and suggestive (1/N) thresholds by Bonferroni correction, in which N is the number of SNPs, were used in the analysis. In this study, the meta-analysis combined the *β*-coefficients and standard errors of the SNPs in the results of single-population GWAS that were common to American and Canadian Duroc pigs.

### Haplotype block analysis

Haplotype block analysis was carried out for chromosomal regions with multiple significant SNPs clustered around the top SNP to evaluate the LD pattern of the regions. The haplotype blocks were generated by the software PLINK v1.9 and Haploview v4.2 [42].

### Estimation of genetic parameters and the explained phenotypic variance

The restricted maximum likelihood method was used to estimate the phenotypic variance explained by the significant SNPs for ADG and LMP traits using GCTA software. To adjust the structure of the stratified population and cryptic relatedness, the top three eigenvectors of PCA were included as covariates in this analysis. The phenotypic variance explained by the significant SNPs were calculated in the following model [39, 43]:

$$ y= X\beta +g+\varepsilon \kern0.4em with\kern0.4em \operatorname{var}(y)={A}_g{\sigma}_g^2+I{\sigma}_{\varepsilon}^2 $$

where *y* refers to the vector of phenotype value; *β* is a vector of fixed effects; *X* is an incidence matrix for *β*; *g* represents the vector of the aggregate effects of all the qualified SNPs for the pigs within one population; *I* is the identity matrix; *A*_*g*_ is the GRM; ${\sigma}_g^2$ corresponds to the additive genetic variance captured by either the genome-wide SNPs or the selected SNPs, and ${\sigma}_{\varepsilon}^2$ refers to the residual variance. GCTA was used to estimate genetic correlation in the bivariate mode, and was also used to compute the genomic heritability.

### Functional annotation of candidate genes

All SNP positions from the most recent *Sus scrofa* genome (version 11.1) were downloaded from Ensembl [44]. The Ensembl annotation of the *Sus scrofa* 11.1 genome version was used to find the genes which were nearest the significant SNPs (http://ensembl.org/Sus_scrofa/Info/Index). KEGG and GO analyses were carried out using the KOBAS 3.0 (http://kobas.cbi.pku.edu.cn/kobas3) [45]. Fisher’s exact test was used to assess the significance of the enriched terms with the criteria of *P* < 0.05 to explore the genes involved in pathways and biological processes [46].

## Supplementary Information

**Additional file 1: Figure S1.** Q–Q plots showing the observed versus expected log *P*-values for ADG and LMP. Q–Q plots showing the observed versus expected log *P*-values for ADG and LMP. The estimated lambda(λ) is shown in the figure. Q–Q plot for (a) Average daily gain (American origin), (b) Average daily gain (Canadian origin), (c) Lean meat percentage (American origin), (d) Lean meat percentage (Canadian origin).

**Additional file 2: Table S1.** Effective SNPs for average daily gain and lean meat percentage traits.

**Additional file 3: Figure S2.** Haplotype block on SSC1 of lean meat percentage on Canadian origin Duroc pigs.

**Additional file 4: Figure S3.** Regional association plot of the significant marker (ASGA0002401) associated with lean meat percentage at SSC1. The plots indicate the association results for LMP on Canadian origin Duroc pigs (a) before and (b) after conditional analysis on ASGA0002401.

**Additional file 5: Table S2.** Significant SNPs and candidate genes for ADG and LMP in meta-analysis.

**Additional file 6: Figure S4.** Venn plot showing relationships of the identified SNPs in this study. ADG: Average daily gain; LMP: Lean meat percentage. (a) Venn plot with showing the shared SNPs by ADG (American origin Duroc), ADG (Canadian origin Duroc), ADG (Meta-analysis), and ADG (Two Duroc populations) (b) Venn plot showing the shared SNPs by LMP (American origin Duroc), LMP (Canadian origin Duroc), LMP (Meta-analysis), and LMP (Two Duroc populations).

**Additional file 7: Table S3.** Comparison of significant SNPs with previously reported QTLs from the pig QTL database.

**Additional file 8: Table S4.** Newly significant SNPs for average daily gain and lean meat percentage.

**Additional file 9: Table S5.** KEGG PATHWAY and GO significant terms with average daily gain trait (*P* < 0.05).

**Additional file 10: Table S6.** KEGG PATHWAY and GO significant terms with lean meat percentage trait (*P* < 0.05).

## Data Availability

The datasets of genotypes analyzed during the current study are available on figshare (10.6084/m9.figshare.8019551.v1). The phenotypic data used in this study are not publicly available since the test populations are consisted of the nucleus herd of Wens Foodstuff Group Co., Ltd., but are available from the corresponding author on reasonable request.

## Abbreviations

ADG: Average daily gain
LMP: Lean meat percentage
CF: Correction factors
BF: Backfat thickness
LMD: Loin muscle depth
PCA: Principal component analysis
Q-Q plot: Quantile-quantile plot
SSC: *Sus scrofa* chromosome
SNP: Single nucleotide polymorphism
GWAS: Genome-wide association study
QTL: Quantitative trait locus
LD: Linkage disequilibrium
GO: Gene ontology
KEGG: Kyoto Encyclopedia of Genes and Genomes
