# Supplementary figures and images for: A meta-analysis of genome-wide association studies for average daily gain and lean meat percentage in two Duroc pig populations
Source: BMC Genomics. 2021 Jan 6;22:12. doi: 10.1186/s12864-020-07288-1 (PMC7788875; doi:10.1186/s12864-020-07288-1)

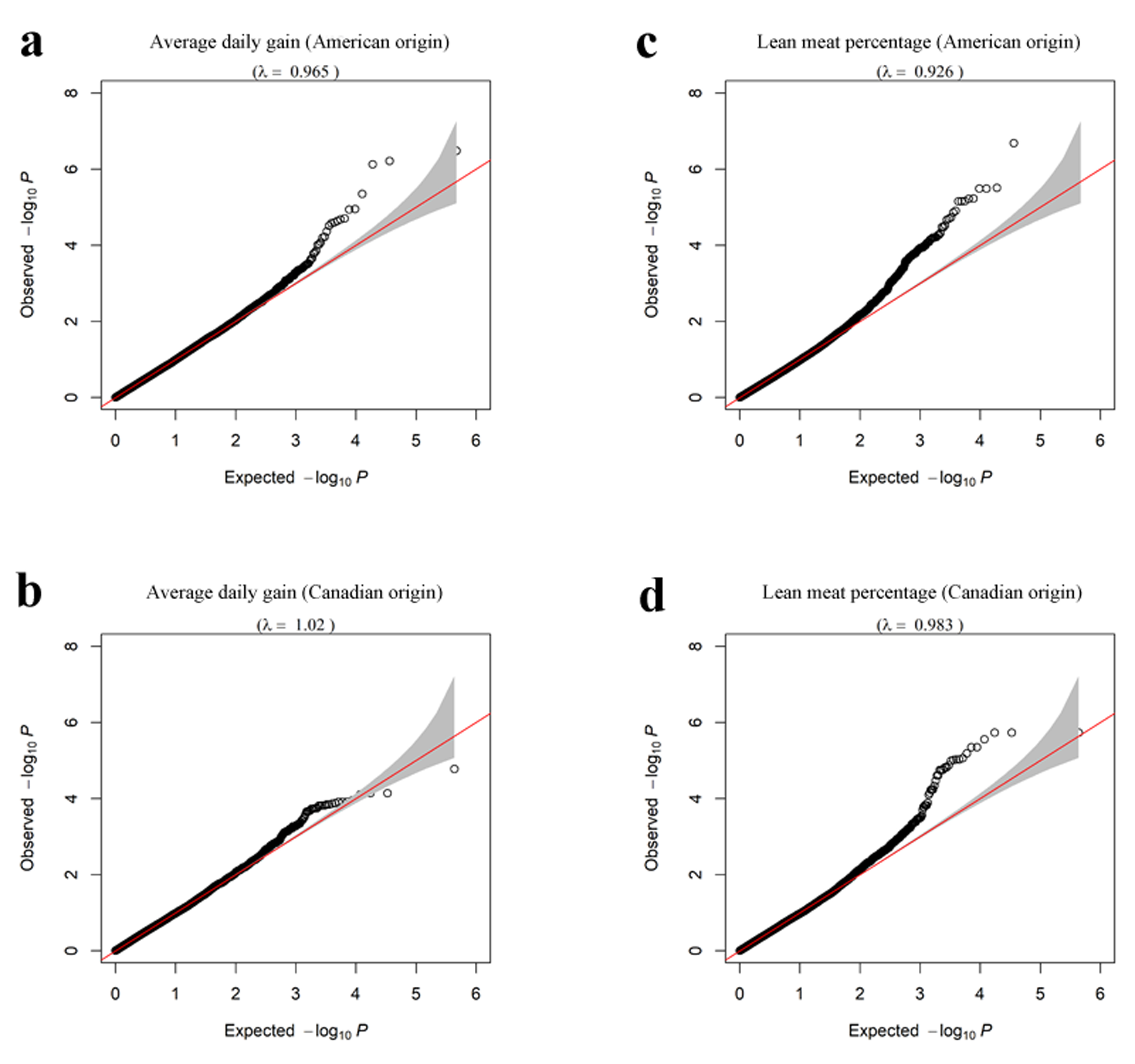

Supplement: Supplementary file 1 — Additional file 1: Figure S1. Q–Q plots showing the observed versus expected log P-values for ADG and LMP. Q–Q plots showing the observed versus expected log P-values for ADG and LMP. The estimated lambda(λ) is shown in the figure. Q–Q plot for (a) Average daily gain (American origin), (b) Average daily gain (Canadian origin), (c) Lean meat percentage (American origin), (d) Lean meat percentage (Canadian origin). [file 12864_2020_7288_MOESM1_ESM.jpg]

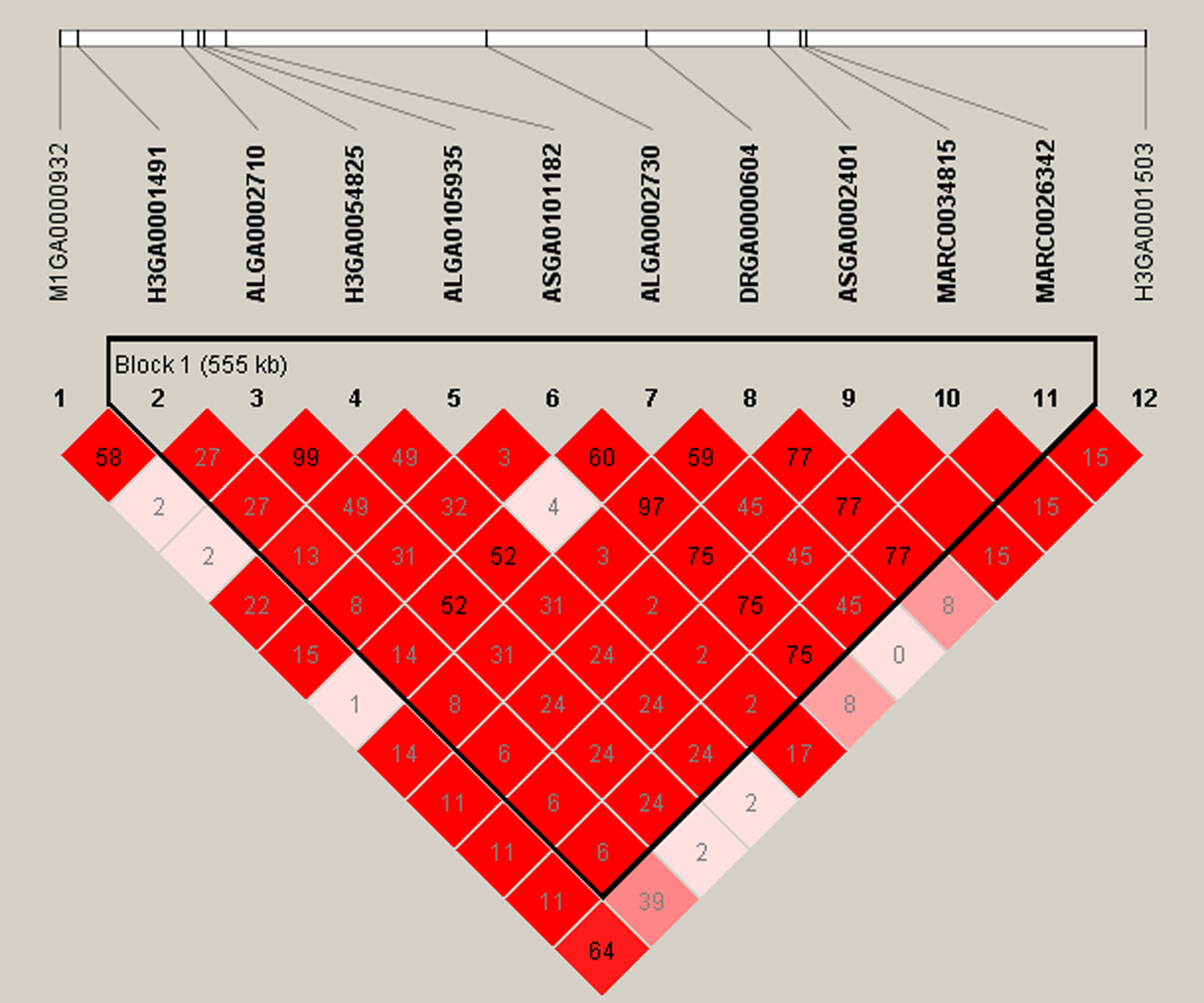

Supplement: Supplementary file 3 — Additional file 3: Figure S2. Haplotype block on SSC1 of lean meat percentage on Canadian origin Duroc pigs. [file 12864_2020_7288_MOESM3_ESM.jpg]

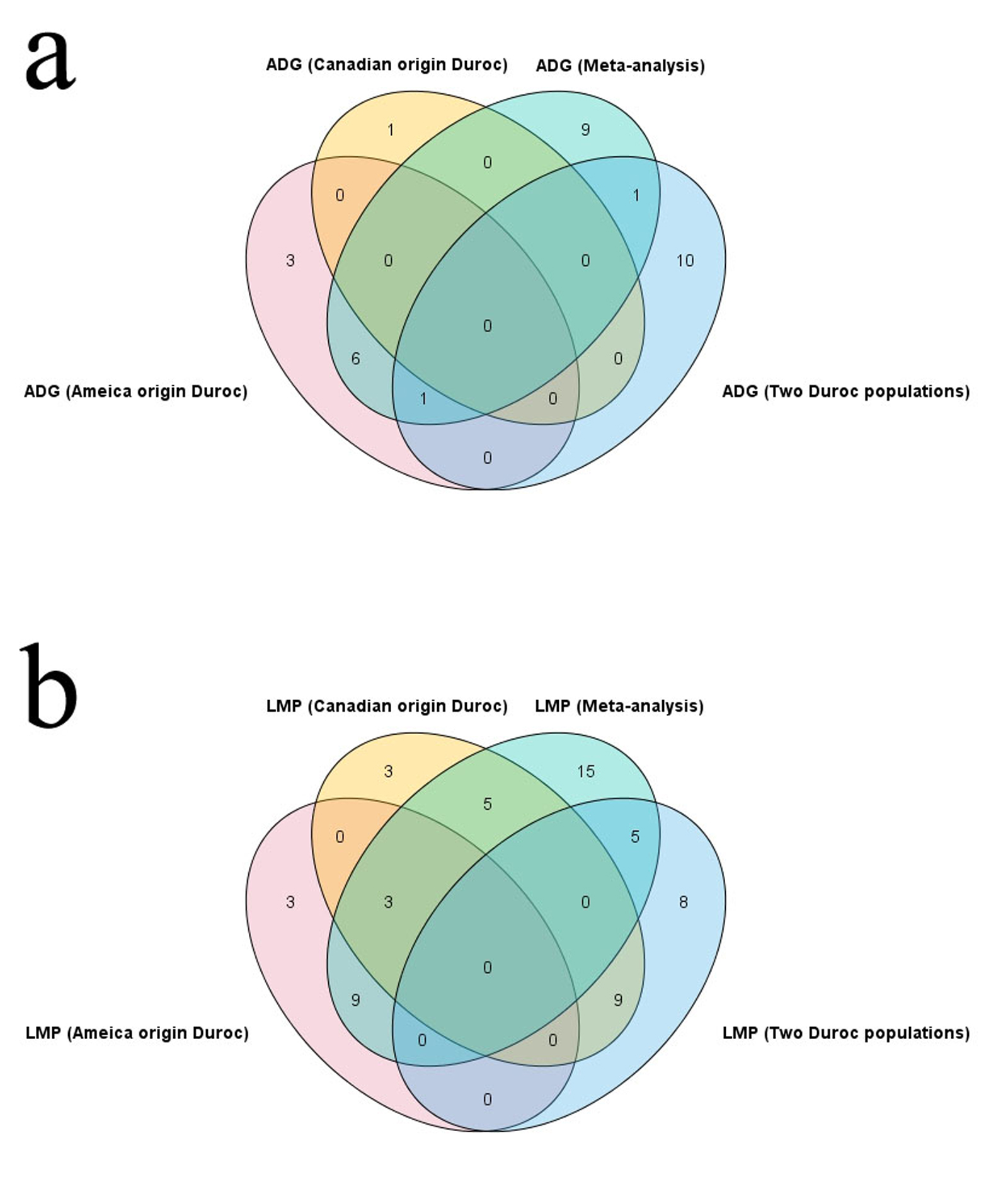

Supplement: Supplementary file 6 — Additional file 6: Figure S4. Venn plot showing relationships of the identified SNPs in this study. ADG: Average daily gain; LMP: Lean meat percentage. (a) Venn plot with showing the shared SNPs by ADG (American origin Duroc), ADG (Canadian origin Duroc), ADG (Meta-analysis), and ADG (Two Duroc populations) (b) Venn plot showing the shared SNPs by LMP (American origin Duroc), LMP (Canadian origin Duroc), LMP (Meta-analysis), and LMP (Two Duroc populations). [file 12864_2020_7288_MOESM6_ESM.jpg]
