# Supplementary material for: A meta-analysis of genome-wide association studies for average daily gain and lean meat percentage in two Duroc pig populations
Source: BMC Genomics. 2021 Jan 6;22:12. doi: 10.1186/s12864-020-07288-1 (PMC7788875; doi:10.1186/s12864-020-07288-1)
Supplement: Supplementary file 2 — Additional file 2: Table S1. Effective SNPs for average daily gain and lean meat percentage traits. [file 12864_2020_7288_MOESM2_ESM.docx]

**Additional file 2: Table S1.** Effective SNPs for average daily gain and lean meat percentage traits.

| Traits | Population^2^ | SNP^2^ | N^3^ |
| --- | --- | --- | --- |
| Average daily gain | AD | 39474 | 3770 |
|  | CD | 36394 | 2090 |
|  | Meta | 35415 | 5860 |
| Lean meat percentage | AD | 39473 | 3769 |
|  | CD | 36393 | 2082 |
|  | Meta | 35412 | 5851 |

^1^AD: American Duroc pig population; CD: Canadian Duroc pig population; Meta: Meta-analysis. ^2^The number of SNPs used in association analysis. ^3^N is number of pigs used in the analysis.
