# Supplementary material for: A meta-analysis of genome-wide association studies for average daily gain and lean meat percentage in two Duroc pig populations
Source: BMC Genomics. 2021 Jan 6;22:12. doi: 10.1186/s12864-020-07288-1 (PMC7788875; doi:10.1186/s12864-020-07288-1)
Supplement: Supplementary file 5 — Additional file 5: Table S2. Significant SNPs and candidate genes for ADG and LMP in meta-analysis. [file 12864_2020_7288_MOESM5_ESM.docx]

**Additional file 5: Table S2.** Significant SNPs and candidate genes for ADG and LMP in meta-analysis.

| Trait^1^ | SSC^2^ | SNP^3^ | Location^4^  (bp) | *P-*value^5^ | Distance^6^  (bp) | Candidate gene |
| --- | --- | --- | --- | --- | --- | --- |
| ADG | 1 | MARC0013872 | 161824864 | **1.28 × 10^-8^** | within | *ENSSSCG00000004911* |
|  | 1 | ASGA0004988 | 159881634 | **1.89 × 10^-8^** | within | *CDH20* |
|  | 1 | ALGA0006623 | 160347188 | **3.22 × 10^-8^** | within | *ENSSSCG00000048538* |
|  | 1 | **WU_10.2_1_178188861** | 160447734 | **3.74 × 10^-8^** | 98849 | *ENSSSCG00000048538* |
|  | 1 | ALGA0006684 | 161853405 | **6.61 × 10^-8^** | within | *ENSSSCG00000004911* |
|  | 1 | H3GA0003149 | 162192627 | **8.91 × 10^-7^** | within | *ALPK2* |
|  | 1 | WU_10.2_1_179575045 | 161987727 | **1.35 × 10^-6^** | within | *ENSSSCG00000004911* |
|  | 1 | **INRA0004898** | 158811662 | 3.81 × 10^-6^ | within | *PHLPP1* |
|  | 1 | **H3GA0003104** | 159619891 | 5.52 × 10^-6^ | -17910 | *RNF152* |
|  | 1 | **ALGA0006736** | 163815021 | 6.09 × 10^-6^ | -24825 | *DENND4A* |
|  | 1 | **MARC0075909** | 159238083 | 6.58 × 10^-6^ | within | *RELCH* |
|  | 1 | **ALGA0006602** | 159538854 | 1.21 × 10^-5^ | within | *RNF152* |
|  | 1 | **ASGA0005079** | 163443518 | 1.32 × 10^-5^ | 3381 | *DPP8* |
|  | 1 | **DRGA0000286** | 23853212 | 1.93 × 10^-5^ | 277587 | *ENSSSCG00000043584* |
|  | 3 | **ASGA0015187** | 76889367 | 1.78 × 10^-5^ | within | *CEP68* |
|  | 7 | **ASGA0032310** | 28584037 | 1.66 × 10^-5^ | within | *PRIM2* |
|  | 14 | WU_10.2_14_8843751 | 7988327 | **1.26 × 10^-6^** | -26761 | *STC1* |
| LMP | 1 | MARC0013872 | 161824864 | **2.86 × 10^-9^** | within | *ENSSSCG00000004911* |
|  | 1 | ALGA0006623 | 160347188 | **1.02 × 10^-8^** | within | *ENSSSCG00000048538* |
|  | 1 | ASGA0004988 | 159881634 | **1.45 × 10^-8^** | within | *CDH20* |
|  | 1 | WU_10.2_1_178188861 | 160447734 | **2.51 × 10^-8^** | 98849 | *ENSSSCG00000048538* |
|  | 1 | ALGA0006602 | 159538854 | **5.44 × 10^-8^** | within | *RNF152* |
|  | 1 | MARC0075909 | 159238083 | **8.17 × 10^-8^** | within | *RELCH* |
|  | 1 | ALGA0006684 | 161853405 | **1.34 × 10^-7^** | within | *ENSSSCG00000004911* |
|  | 1 | H3GA0003104 | 159619891 | **1.36 × 10^-7^** | -17910 | *RNF152* |
|  | 1 | INRA0004898 | 158811662 | **2.11 × 10^-7^** | within | *PHLPP1* |
|  | 1 | **MARC0034873** | 158682904 | **1.19 × 10^-6^** | within | *PHLPP1* |
|  | 1 | **INRA0004895** | 158755255 | **1.21 × 10^-6^** | within | *PHLPP1* |
|  | 1 | WU_10.2_1_179575045 | 161987727 | **1.24 × 10^-6^** | within | *ENSSSCG00000004911* |
|  | 1 | H3GA0003149 | 162192627 | 1.94 × 10^-6^ | within | *ALPK2* |
|  | 1 | ALGA0123800 | 254207127 | 2.79 × 10^-6^ | within | *RGS3* |
|  | 1 | **ASGA0004922** | 151018802 | 8.19 × 10^-6^ | -38045 | *CBLN2* |
|  | 1 | **WU_10.2_1_40430395** | 36657204 | 2.50 × 10^-5^ | within | *CENPW* |
|  | 2 | **WU_10.2_2_76986997** | 76440900 | 2.91 × 10^-6^ | within | *AP3D1* |
|  | 2 | 10006986 | 76416246 | 3.86 × 10^-6^ | within | *AMH* |
|  | 2 | **DIAS0000957** | 41991741 | 4.92 × 10^-6^ | within | *PIK3C2A* |
|  | 2 | WU_10.2_2_82907810 | 81306158 | 5.54 × 10^-6^ | -3295 | *SNCB* |
|  | 2 | **ASGA0010206** | 41793635 | 5.83 × 10^-6^ | within | *ENSSSCG00000013380* |
|  | 2 | **ASGA0010202** | 41830637 | 6.55 × 10^-6^ | -103 | *ENSSSCG00000013380* |
|  | 2 | **WU_10.2_2_78408183** | 77599286 | 7.51 × 10^-6^ | 2856 | *MISP* |
|  | 2 | **DRGA0002970** | 42362844 | 7.55 × 10^-6^ | within | *C11orf58* |
|  | 2 | **WU_10.2_2_81039039** | 79435374 | 8.00 × 10^-6^ | within | *ZNF879* |
|  | 2 | **WU_10.2_2_77233544** | 76535013 | 1.61 × 10^-5^ | 3486 | *BTBD2* |
|  | 2 | **H3GA0052833** | 76599983 | 1.79 × 10^-5^ | within | *CSNK1G2* |
|  | 2 | **WU_10.2_2_80933825** | 79330159 | 1.89 × 10^-5^ | within | *ADAMTS2* |
|  | 2 | **WU_10.2_2_76564291** | 76013400 | 1.93 × 10^-5^ | -84248 | *DIRAS1* |
|  | 2 | **WU_10.2_2_79336306** | 77997390 | 1.99 × 10^-5^ | 22196 | *ENSSSCG00000044074* |
|  | 2 | **WU_10.2_2_79760743** | 78275714 | 2.28 × 10^-5^ | 9489 | *GFPT2* |
|  | 2 | **ALGA0102692** | 77362555 | 2.38 × 10^-5^ | within | *ABCA7* |
|  | 2 | **WU_10.2_2_78173959** | 76905754 | 2.47 × 10^-5^ | within | *ENSSSCG00000013436* |
|  | 6 | ASGA0096606 | 48241180 | 2.44 × 10^-5^ | 31313 | *LGALS13* |
|  | 7 | **WU_10.2_7_707738** | 651731 | 2.27 × 10^-5^ | 12642 | *FOXQ1* |
|  | 12 | ALGA0105911 | 27109190 | 2.20 × 10^-5^ | -8767 | *WFIKKN2* |
|  | 15 | WU_10.2_15_156432561 | 57333035 | 2.95 × 10^-6^ | within | *ARHGEF4* |

^1^Average daily gain (ADG), lean meat percentage (LMP). ^2^*Sus scrofa* chromosome (SSC). ^3^SNP ID in boldface: these SNPs are meta-analysis identified additional SNP. ^4^SNP positions in Ensembl. ^5^*P*-value in boldface: genome-wide significant; *P*-value not in boldface: suggestive significant. ^6^+/−: the SNP located upstream/downstream of the nearest gene.
