# Supplementary material for: A meta-analysis of genome-wide association studies for average daily gain and lean meat percentage in two Duroc pig populations
Source: BMC Genomics. 2021 Jan 6;22:12. doi: 10.1186/s12864-020-07288-1 (PMC7788875; doi:10.1186/s12864-020-07288-1)
Supplement: Supplementary file 7 — Additional file 7: Table S3. Comparison of significant SNPs with previously reported QTLs from the pig QTL database. [file 12864_2020_7288_MOESM7_ESM.docx]

**Additional file 7: Table S3.** Comparison of significant SNPs with previously reported QTLs from the pig QTL database.

| Trait^1^ | SSC^2^ | SNP | Location^3^  (bp) | Population^4^ | Starting QTL position (bp)^5^ | Ending QTL position (bp)^6^ | ID^7^ | Corresponded trait in the QTL database |
| --- | --- | --- | --- | --- | --- | --- | --- | --- |
| ADG | 1 | MARC0013872 | 161824864 | AD & Meta | 62261447 | 162073157 | 329 | ADG |
|  | 1 | ASGA0004988 | 159881634 | AD & Meta | 62261447 | 162073157 | 329 | ADG |
|  | 1 | ALGA0006623 | 160347188 | AD & Meta | 62261447 | 162073157 | 329 | ADG |
|  | 1 | WU_10.2_1_178188861 | 160447734 | Meta | 62261447 | 162073157 | 329 | ADG |
|  | 1 | ALGA0006684 | 161853405 | AD & Meta | 62261447 | 162073157 | 329 | ADG |
|  | 1 | ALGA0006736 | 163815021 | Meta | 162073157 | 233806417 | 140 | ADG |
|  | 1 | H3GA0003149 | 162192627 | AD & Meta | 162073157 | 233806417 | 140 | ADG |
|  | 1 | ASGA0005079 | 163443518 | Meta | 162073157 | 233806417 | 140 | ADG |
|  | 1 | INRA0004898 | 158811662 | Meat | 62261447 | 162073157 | 329 | ADG |
|  | 1 | WU_10.2_1_179575045 | 161987727 | AD & Meta | 62261447 | 162073157 | 329 | ADG |
|  | 1 | H3GA0003104 | 159619891 | Meta | 62261447 | 162073157 | 329 | ADG |
|  | 1 | MARC0075909 | 159238083 | Meta | 62261447 | 162073157 | 329 | ADG |
|  | 1 | ALGA0006602 | 159538854 | Meta | 62261447 | 162073157 | 329 | ADG |
|  | 1 | DRGA0000286 | 23853212 | Meta | 20064508 | 36144885 | 338 | ADG |
|  | 1 | ASGA0004970 | 158589475 | CD | 62261447 | 162073157 | 329 | ADG |
|  | 3 | ASGA0015187 | 76889367 | Meta | 34143639 | 98066155 | 173 | ADG |
|  | 7 | ASGA0032310 | 28584037 | Meta | 48748 | 120853004 | 3770 | ADG |
|  | 8 | DIAS0000782 | 30256709 | AD | 29785347 | 34089646 | 64 | ADG |
| LMP | 1 | ALGA0123800 | 254207127 | AD & Meta | 220801047 | 266165650 | 1264 | LMP |
|  | 2 | DIAS0000957 | 41991741 | Meta | 21136167 | 44483418 | 897 | LMP |
|  | 2 | DRGA0002970 | 42362844 | Meta | 21136167 | 44483418 | 897 | LMP |
|  | 2 | ASGA0010206 | 41793635 | Meta | 21136167 | 44483418 | 897 | LMP |
|  | 2 | ASGA0010202 | 41830637 | Meta | 21136167 | 44483418 | 897 | LMP |
|  | 6 | ASGA0096606 | 48241180 | AD & Meta | 45448178 | 77813565 | 996 | LMP |
|  | 6 | ASGA0091829 | 48289460 | AD | 45448178 | 77813565 | 996 | LMP |
|  | 6 | WU_10.2_6_41924003 | 46461817 | AD | 45448178 | 77813565 | 996 | LMP |
|  | 7 | WU_10.2_7_707738 | 651731 | Meta | 48748 | 120853004 | 3769 | LMP |
|  | 15 | WU_10.2_15_156432561 | 57333035 | AD & Meta | 25021683 | 64824079 | 1172 | LMP |

^1^Average daily gain (ADG), lean meat percentage (LMP). ^2^*Sus scrofa* chromosome (SSC). ^3^SNP positions in Ensembl. ^4^American Duroc pig (AD), Canadian Duroc pig (CD), Meta-analysis (Meta). ^5^Starting position of the mapped QTL in the QTL database. ^6^Ending position of the mapped QTL in the QTL database. ^7^Identity of QTL in the pig QTL database.
