# Supplementary material for: A meta-analysis of genome-wide association studies for average daily gain and lean meat percentage in two Duroc pig populations
Source: BMC Genomics. 2021 Jan 6;22:12. doi: 10.1186/s12864-020-07288-1 (PMC7788875; doi:10.1186/s12864-020-07288-1)
Supplement: Supplementary file 8 — Additional file 8: Table S4. Newly significant SNPs for average daily gain and lean meat percentage. [file 12864_2020_7288_MOESM8_ESM.docx]

**Additional file 8: Table S4.** Newly significant SNPs for average daily gain and lean meat percentage

| Trait^1^ | SSC^2^ | SNP | Location^3^ (bp) | Population^4^ |
| --- | --- | --- | --- | --- |
| ADG | 6 | Affx-114594216 | 168268278 | AD |
|  | 11 | WU_10.2_11_86301815 | 78432491 | AD |
|  | 14 | WU_10.2_14_8843751 | 7988327 | AD & Meta |
| LMP | 1 | WU_10.2_1_40430395 | 36657204 | CD |
|  | 1 | H3GA0001466 | 37024102 | CD |
|  | 1 | H3GA0001475 | 37366714 | CD |
|  | 1 | DRGA0000591 | 37381311 | CD |
|  | 1 | MARC0114211 | 37401594 | CD |
|  | 1 | ASGA0101182 | 37746276 | CD |
|  | 1 | DRGA0000604 | 38067414 | CD |
|  | 1 | ASGA0002401 | 38161769 | CD |
|  | 1 | MARC0034815 | 38185044 | CD |
|  | 1 | MARC0026342 | 38189919 | CD |
|  | 1 | ASGA0004922 | 151018802 | Meta |
|  | 1 | MARC0034873 | 158682904 | Meta |
|  | 1 | INRA0004895 | 158755255 | Meta |
|  | 1 | INRA0004898 | 158811662 | CD & Meta |
|  | 1 | MARC0075909 | 159238083 | CD & Meta |
|  | 1 | ALGA0006602 | 159538854 | CD & Meta |
|  | 1 | H3GA0003104 | 159619891 | CD & Meta |
|  | 1 | ASGA0004988 | 159881634 | AD & CD & Meta |
|  | 1 | ALGA0006623 | 160347188 | AD & CD & Meta |
|  | 1 | WU_10.2_1_178188861 | 160447734 | CD & Meta |
|  | 1 | MARC0013872 | 161824864 | AD & CD & Meta |
|  | 1 | ALGA0006684 | 161853405 | AD & Meta |
|  | 1 | WU_10.2_1_179575045 | 161987727 | AD & Meta |
|  | 1 | H3GA0003149 | 162192627 | AD & Meta |
|  | 2 | WU_10.2_2_76564291 | 76013400 | Meta |
|  | 2 | 10006986 | 76416246 | AD & Meta |
|  | 2 | WU_10.2_2_76986997 | 76440900 | Meta |
|  | 2 | WU_10.2_2_77233544 | 76535013 | Meta |
|  | 2 | H3GA0052833 | 76599983 | Meta |
|  | 2 | WU_10.2_2_78173959 | 76905754 | Meta |
|  | 2 | ALGA0102692 | 77362555 | Meta |
|  | 2 | WU_10.2_2_78408183 | 77599286 | Meta |
|  | 2 | WU_10.2_2_79336306 | 77997390 | Meta |
|  | 2 | WU_10.2_2_79760743 | 78275714 | Meta |
|  | 2 | WU_10.2_2_80933825 | 79330159 | Meta |
|  | 2 | WU_10.2_2_81039039 | 79435374 | Meta |
|  | 2 | WU_10.2_2_82907810 | 81306158 | AD & Meta |
|  | 4 | ASGA0020293 | 74693408 | CD |
|  | 4 | H3GA0013036 | 74714319 | CD |
|  | 4 | MARC0093868 | 74774997 | CD |
|  | 6 | Affx-114594216 | 168268278 | AD |
|  | 12 | ALGA0105911 | 27109190 | AD & Meta |

^1^ADG: average daily gain; LMP: lean meat percentage. ^2^SSC: *Sus scrofa* chromosome. ^3^SNP positions in Ensembl. ^4^AD: American Duroc pig population; CD: Canadian Duroc pig population; Meta: Meta-analysis.
