# Supplementary material for: A meta-analysis of genome-wide association studies for average daily gain and lean meat percentage in two Duroc pig populations
Source: BMC Genomics. 2021 Jan 6;22:12. doi: 10.1186/s12864-020-07288-1 (PMC7788875; doi:10.1186/s12864-020-07288-1)
Supplement: Supplementary file 9 — Additional file 9: Table S5. KEGG PATHWAY and GO significant terms with average daily gain trait (P < 0.05). [file 12864_2020_7288_MOESM9_ESM.docx]

**Additional file 9: Table S5.** KEGG PATHWAY and GO significant terms with average daily gain trait (*P* < 0.05).

| Term | Database | ID | Gene names | *P*-Value |
| --- | --- | --- | --- | --- |
| bone growth | Gene Ontology | GO:0098868 | *STC1* | 0.002243451 |
| cartilage development involved in endochondral bone morphogenesis | Gene Ontology | GO:0060351 | *STC1* | 0.002243451 |
| excretion | Gene Ontology | GO:0007588 | *STC1* | 0.002616919 |
| positive regulation of calcium ion import | Gene Ontology | GO:0090280 | *STC1* | 0.002616919 |
| chondrocyte proliferation | Gene Ontology | GO:0035988 | *STC1* | 0.002616919 |
| regulation of cardiac muscle cell contraction | Gene Ontology | GO:0086004 | *STC1* | 0.002616919 |
| negative regulation of calcium ion transport | Gene Ontology | GO:0051926 | *STC1* | 0.002990261 |
| regulation of calcium ion import | Gene Ontology | GO:0090279 | *STC1* | 0.002990261 |
| regulation of actin filament-based movement | Gene Ontology | GO:1903115 | *STC1* | 0.002990261 |
| negative regulation of endothelial cell migration | Gene Ontology | GO:0010596 | *STC1* | 0.003736567 |
| endochondral bone morphogenesis | Gene Ontology | GO:0060350 | *STC1* | 0.004109532 |
| cardiac muscle cell contraction | Gene Ontology | GO:0086003 | *STC1* | 0.004109532 |
| calcium ion import | Gene Ontology | GO:0070509 | *STC1* | 0.004109532 |
| negative regulation of epithelial cell migration | Gene Ontology | GO:0010633 | *STC1* | 0.004482371 |
| actin-mediated cell contraction | Gene Ontology | GO:0070252 | *STC1* | 0.005227673 |
| endothelial cell development | Gene Ontology | GO:0001885 | *STC1* | 0.005972472 |
| regulation of cardiac muscle contraction | Gene Ontology | GO:0055117 | *STC1* | 0.006344683 |
| renal system process | Gene Ontology | GO:0003014 | *STC1* | 0.007088729 |
| actin filament-based movement | Gene Ontology | GO:0030048 | *STC1* | 0.007088729 |
| regulation of striated muscle contraction | Gene Ontology | GO:0006942 | *STC1* | 0.007460564 |
| bone morphogenesis | Gene Ontology | GO:0060349 | *STC1* | 0.007832274 |
| regulation of anion transport | Gene Ontology | GO:0044070 | *STC1* | 0.008203859 |
| negative regulation of ion transport | Gene Ontology | GO:0043271 | *STC1* | 0.008203859 |
| axis specification | Gene Ontology | GO:0009798 | *STC1* | 0.008203859 |
| regulation of endothelial cell migration | Gene Ontology | GO:0010594 | *STC1* | 0.008575318 |
| organ growth | Gene Ontology | GO:0035265 | *STC1* | 0.009317861 |
| cardiac muscle contraction | Gene Ontology | GO:0060048 | *STC1* | 0.010059903 |
| positive regulation of calcium ion transport | Gene Ontology | GO:0051928 | *STC1* | 0.010059903 |
| endothelial cell migration | Gene Ontology | GO:0043542 | *STC1* | 0.010801445 |
| endothelial cell differentiation | Gene Ontology | GO:0045446 | *STC1* | 0.010801445 |
| endothelium development | Gene Ontology | GO:0003158 | *STC1* | 0.011542487 |
| bone development | Gene Ontology | GO:0060348 | *STC1* | 0.012653113 |
| regulation of epithelial cell migration | Gene Ontology | GO:0010632 | *STC1* | 0.012653113 |
| striated muscle contraction | Gene Ontology | GO:0006941 | *STC1* | 0.012653113 |
| regulation of muscle contraction | Gene Ontology | GO:0006937 | *STC1* | 0.013023073 |
| regulation of heart contraction | Gene Ontology | GO:0008016 | *STC1* | 0.013392907 |
| negative regulation of cell motility | Gene Ontology | GO:2000146 | *STC1* | 0.014870997 |
| negative regulation of cell migration | Gene Ontology | GO:0030336 | *STC1* | 0.014870997 |
| epithelium migration | Gene Ontology | GO:0090132 | *STC1* | 0.015609295 |
| epithelial cell migration | Gene Ontology | GO:0010631 | *STC1* | 0.015609295 |
| regulation of muscle system process | Gene Ontology | GO:0090257 | *STC1* | 0.015978257 |
| heart contraction | Gene Ontology | GO:0060047 | *STC1* | 0.015978257 |
| heart process | Gene Ontology | GO:0003015 | *STC1* | 0.016347095 |
| positive regulation of ion transport | Gene Ontology | GO:0043270 | *STC1* | 0.016715808 |
| tissue migration | Gene Ontology | GO:0090130 | *STC1* | 0.016715808 |
| regulation of calcium ion transport | Gene Ontology | GO:0051924 | *STC1* | 0.017084396 |
| cartilage development | Gene Ontology | GO:0051216 | *STC1* | 0.017452861 |
| epithelial cell development | Gene Ontology | GO:0002064 | *STC1* | 0.017452861 |
| negative regulation of cellular component movement | Gene Ontology | GO:0051271 | *STC1* | 0.018189417 |
| negative regulation of locomotion | Gene Ontology | GO:0040013 | *STC1* | 0.018557508 |
| regulation of blood circulation | Gene Ontology | GO:1903522 | *STC1* | 0.018925476 |
| skeletal system morphogenesis | Gene Ontology | GO:0048705 | *STC1* | 0.020763451 |
| connective tissue development | Gene Ontology | GO:0061448 | *STC1* | 0.021497772 |
| regulation of actin filament-based process | Gene Ontology | GO:0032970 | *STC1* | 0.022964929 |
| muscle contraction | Gene Ontology | GO:0006936 | *STC1* | 0.023331409 |
| hormone activity | Gene Ontology | GO:0005179 | *STC1* | 0.023331409 |
| ameboidal-type cell migration | Gene Ontology | GO:0001667 | *STC1* | 0.024796091 |
| regulation of metal ion transport | Gene Ontology | GO:0010959 | *STC1* | 0.024796091 |
| cellular calcium ion homeostasis | Gene Ontology | GO:0006874 | *STC1* | 0.026258796 |
| calcium ion homeostasis | Gene Ontology | GO:0055074 | *STC1* | 0.026258796 |
| muscle system process | Gene Ontology | GO:0003012 | *STC1* | 0.026624164 |
| cellular divalent inorganic cation homeostasis | Gene Ontology | GO:0072503 | *STC1* | 0.028084401 |
| calcium ion transport | Gene Ontology | GO:0006816 | *STC1* | 0.028084401 |
| divalent inorganic cation homeostasis | Gene Ontology | GO:0072507 | *STC1* | 0.028449152 |
| blood circulation | Gene Ontology | GO:0008015 | *STC1* | 0.031362728 |
| divalent inorganic cation transport | Gene Ontology | GO:0072511 | *STC1* | 0.031726372 |
| divalent metal ion transport | Gene Ontology | GO:0070838 | *STC1* | 0.031726372 |
| circulatory system process | Gene Ontology | GO:0003013 | *STC1* | 0.032089892 |
| negative regulation of transport | Gene Ontology | GO:0051051 | *STC1* | 0.033542747 |
| cellular metal ion homeostasis | Gene Ontology | GO:0006875 | *STC1* | 0.034993639 |
| pattern specification process | Gene Ontology | GO:0007389 | *STC1* | 0.035718349 |
| regulation of system process | Gene Ontology | GO:0044057 | *STC1* | 0.036804496 |
| cellular cation homeostasis | Gene Ontology | GO:0030003 | *STC1* | 0.03825098 |
| skeletal system development | Gene Ontology | GO:0001501 | *STC1* | 0.038612295 |
| metal ion homeostasis | Gene Ontology | GO:0055065 | *STC1* | 0.038973488 |
| cellular ion homeostasis | Gene Ontology | GO:0006873 | *STC1* | 0.038973488 |
| cell morphogenesis involved in differentiation | Gene Ontology | GO:0000904 | *STC1* | 0.042218735 |
| developmental growth | Gene Ontology | GO:0048589 | *STC1* | 0.04293856 |
| regulation of ion transport | Gene Ontology | GO:0043269 | *STC1* | 0.04329829 |
| cation homeostasis | Gene Ontology | GO:0055080 | *STC1* | 0.043657898 |
| inorganic ion homeostasis | Gene Ontology | GO:0098771 | *STC1* | 0.044017384 |
| epithelial cell differentiation | Gene Ontology | GO:0030855 | *STC1* | 0.044017384 |
| anion transport | Gene Ontology | GO:0006820 | *STC1* | 0.045095114 |
| regulation of cell migration | Gene Ontology | GO:0030334 | *STC1* | 0.046171751 |
| actin filament-based process | Gene Ontology | GO:0030029 | *STC1* | 0.046530387 |
| ion homeostasis | Gene Ontology | GO:0050801 | *STC1* | 0.049037443 |
| regulation of cell motility | Gene Ontology | GO:2000145 | *STC1* | 0.049395109 |
