# Supplementary material for: A meta-analysis of genome-wide association studies for average daily gain and lean meat percentage in two Duroc pig populations
Source: BMC Genomics. 2021 Jan 6;22:12. doi: 10.1186/s12864-020-07288-1 (PMC7788875; doi:10.1186/s12864-020-07288-1)
Supplement: Supplementary file 10 — Additional file 10: Table S6. KEGG PATHWAY and GO significant terms with lean meat percentage trait (P < 0.05). [file 12864_2020_7288_MOESM10_ESM.docx]

**Additional file 10: Table S6.** KEGG PATHWAY and GO significant terms with lean meat percentage trait (*P* < 0.05).

| Term | Database | ID | Gene names | *P*-Value |
| --- | --- | --- | --- | --- |
| Alanine, aspartate and glutamate metabolism | KEGG PATHWAY | ssc00250 | *GFPT2* | 0.040421407 |
| Hedgehog signaling pathway | KEGG PATHWAY | ssc04340 | *CSNK1G2* | 0.04769335 |
| striated muscle cell differentiation | Gene Ontology | GO:0051146 | *DYRK1B, HEY2* | 0.001807748 |
| carbohydrate binding | Gene Ontology | GO:0030246 | *LGALS13, GFPT2* | 0.001869922 |
| muscle cell differentiation | Gene Ontology | GO:0042692 | *DYRK1B, HEY2* | 0.003000367 |
| regulation of apoptotic process | Gene Ontology | GO:0042981 | *LGALS13, SNCB, HEY2* | 0.003853085 |
| regulation of programmed cell death | Gene Ontology | GO:0043067 | *LGALS13, SNCB, HEY2* | 0.003927102 |
| regulation of cell death | Gene Ontology | GO:0010941 | *LGALS13, SNCB, HEY2* | 0.004591548 |
| semi-lunar valve development | Gene Ontology | GO:1905314 | *HEY2* | 0.006489851 |
| Sin3 complex | Gene Ontology | GO:0016580 | *HEY2* | 0.006489851 |
| UDP-N-acetylglucosamine metabolic process | Gene Ontology | GO:0006047 | *GFPT2* | 0.006489851 |
| apoptotic process | Gene Ontology | GO:0006915 | *LGALS13, SNCB, HEY2* | 0.006513097 |
| programmed cell death | Gene Ontology | GO:0012501 | *LGALS13, SNCB, HEY2* | 0.006769245 |
| heart valve formation | Gene Ontology | GO:0003188 | *HEY2* | 0.007567532 |
| ventricular cardiac muscle tissue morphogenesis | Gene Ontology | GO:0055010 | *HEY2* | 0.007567532 |
| coronary vasculature development | Gene Ontology | GO:0060976 | *HEY2* | 0.007567532 |
| cardiac right ventricle morphogenesis | Gene Ontology | GO:0003215 | *HEY2* | 0.007567532 |
| positive regulation of lymphocyte apoptotic process | Gene Ontology | GO:0070230 | *LGALS13* | 0.007567532 |
| labyrinthine layer blood vessel development | Gene Ontology | GO:0060716 | *HEY2* | 0.007567532 |
| positive regulation of heart rate | Gene Ontology | GO:0010460 | *HEY2* | 0.007567532 |
| ventricular septum morphogenesis | Gene Ontology | GO:0060412 | *HEY2* | 0.007567532 |
| regulation of vasculogenesis | Gene Ontology | GO:2001212 | *HEY2* | 0.007567532 |
| regulation of transcription initiation from RNA polymerase II promoter | Gene Ontology | GO:0060260 | *HEY2* | 0.007567532 |
| atrioventricular valve morphogenesis | Gene Ontology | GO:0003181 | *HEY2* | 0.007567532 |
| trabecula morphogenesis | Gene Ontology | GO:0061383 | *HEY2* | 0.007567532 |
| vascular smooth muscle cell differentiation | Gene Ontology | GO:0035886 | *HEY2* | 0.007567532 |
| cell death | Gene Ontology | GO:0008219 | *LGALS13, SNCB, HEY2* | 0.007853788 |
| muscle structure development | Gene Ontology | GO:0061061 | *DYRK1B, HEY2* | 0.008481699 |
| cardiac muscle cell proliferation | Gene Ontology | GO:0060038 | *HEY2* | 0.008644084 |
| positive regulation of cardiac muscle tissue growth | Gene Ontology | GO:0055023 | *HEY2* | 0.008644084 |
| regulation of cardiac muscle cell proliferation | Gene Ontology | GO:0060043 | *HEY2* | 0.008644084 |
| cardiac muscle tissue growth | Gene Ontology | GO:0055017 | *HEY2* | 0.008644084 |
| adipose tissue development | Gene Ontology | GO:0060612 | *DYRK1B* | 0.008644084 |
| placenta blood vessel development | Gene Ontology | GO:0060674 | *HEY2* | 0.008644084 |
| muscle cell apoptotic process | Gene Ontology | GO:0010657 | *HEY2* | 0.008644084 |
| positive regulation of cardiac muscle cell proliferation | Gene Ontology | GO:0060045 | *HEY2* | 0.008644084 |
| regulation of cardiac muscle tissue growth | Gene Ontology | GO:0055021 | *HEY2* | 0.008644084 |
| regulation of muscle cell apoptotic process | Gene Ontology | GO:0010660 | *HEY2* | 0.008644084 |
| striated muscle hypertrophy | Gene Ontology | GO:0014897 | *HEY2* | 0.008644084 |
| cardiac muscle hypertrophy | Gene Ontology | GO:0003300 | *HEY2* | 0.008644084 |
| positive regulation of heart contraction | Gene Ontology | GO:0045823 | *HEY2* | 0.008644084 |
| muscle hypertrophy | Gene Ontology | GO:0014896 | *HEY2* | 0.008644084 |
| ventricular cardiac muscle tissue development | Gene Ontology | GO:0003229 | *HEY2* | 0.008644084 |
| atrioventricular valve development | Gene Ontology | GO:0003171 | *HEY2* | 0.009719509 |
| positive regulation of leukocyte apoptotic process | Gene Ontology | GO:2000108 | *LGALS13* | 0.009719509 |
| striated muscle adaptation | Gene Ontology | GO:0014888 | *HEY2* | 0.009719509 |
| atrial septum morphogenesis | Gene Ontology | GO:0060413 | *HEY2* | 0.009719509 |
| Sin3-type complex | Gene Ontology | GO:0070822 | *HEY2* | 0.009719509 |
| transaminase activity | Gene Ontology | GO:0008483 | *GFPT2* | 0.009719509 |
| atrial septum development | Gene Ontology | GO:0003283 | *HEY2* | 0.009719509 |
| regulation of DNA-templated transcription, initiation | Gene Ontology | GO:2000142 | *HEY2* | 0.009719509 |
| aorta morphogenesis | Gene Ontology | GO:0035909 | *HEY2* | 0.009719509 |
| inner ear auditory receptor cell differentiation | Gene Ontology | GO:0042491 | *HEY2* | 0.009719509 |
| dopamine metabolic process | Gene Ontology | GO:0042417 | *SNCB* | 0.009719509 |
| negative regulation of transcription regulatory region DNA binding | Gene Ontology | GO:2000678 | *HEY2* | 0.009719509 |
| positive regulation of heart growth | Gene Ontology | GO:0060421 | *HEY2* | 0.009719509 |
| regulation of epidermal cell differentiation | Gene Ontology | GO:0045604 | *HEY2* | 0.009719509 |
| regulation of heart growth | Gene Ontology | GO:0060420 | *HEY2* | 0.009719509 |
| heart growth | Gene Ontology | GO:0060419 | *HEY2* | 0.010793807 |
| hair cell differentiation | Gene Ontology | GO:0035315 | *HEY2* | 0.010793807 |
| cardiac epithelial to mesenchymal transition | Gene Ontology | GO:0060317 | *HEY2* | 0.010793807 |
| nucleotide-sugar metabolic process | Gene Ontology | GO:0009225 | *GFPT2* | 0.010793807 |
| aorta development | Gene Ontology | GO:0035904 | *HEY2* | 0.010793807 |
| cardiac atrium development | Gene Ontology | GO:0003230 | *HEY2* | 0.011866979 |
| regulation of cardiac muscle tissue development | Gene Ontology | GO:0055024 | *HEY2* | 0.011866979 |
| transferase activity, transferring nitrogenous groups | Gene Ontology | GO:0016769 | *GFPT2* | 0.011866979 |
| cardiac atrium morphogenesis | Gene Ontology | GO:0003209 | *HEY2* | 0.011866979 |
| cardiac muscle tissue morphogenesis | Gene Ontology | GO:0055008 | *HEY2* | 0.011866979 |
| positive regulation of cardiac muscle tissue development | Gene Ontology | GO:0055025 | *HEY2* | 0.011866979 |
| heart valve morphogenesis | Gene Ontology | GO:0003179 | *HEY2* | 0.012939028 |
| inner ear receptor cell differentiation | Gene Ontology | GO:0060113 | *HEY2* | 0.012939028 |
| ventricular septum development | Gene Ontology | GO:0003281 | *HEY2* | 0.012939028 |
| neuroepithelial cell differentiation | Gene Ontology | GO:0060563 | *HEY2* | 0.012939028 |
| muscle adaptation | Gene Ontology | GO:0043500 | *HEY2* | 0.012939028 |
| catechol-containing compound metabolic process | Gene Ontology | GO:0009712 | *SNCB* | 0.012939028 |
| cardiac muscle cell development | Gene Ontology | GO:0055013 | *HEY2* | 0.012939028 |
| RNA polymerase II activating transcription factor binding | Gene Ontology | GO:0001102 | *HEY2* | 0.012939028 |
| striated muscle cell proliferation | Gene Ontology | GO:0014855 | *HEY2* | 0.012939028 |
| negative regulation of Notch signaling pathway | Gene Ontology | GO:0045746 | *HEY2* | 0.012939028 |
| catecholamine metabolic process | Gene Ontology | GO:0006584 | *SNCB* | 0.012939028 |
| artery morphogenesis | Gene Ontology | GO:0048844 | *HEY2* | 0.012939028 |
| mechanoreceptor differentiation | Gene Ontology | GO:0042490 | *HEY2* | 0.014009953 |
| cochlea development | Gene Ontology | GO:0090102 | *HEY2* | 0.014009953 |
| regulation of T cell apoptotic process | Gene Ontology | GO:0070232 | *LGALS13* | 0.014009953 |
| cardiac cell development | Gene Ontology | GO:0055006 | *HEY2* | 0.014009953 |
| heart valve development | Gene Ontology | GO:0003170 | *HEY2* | 0.014009953 |
| positive regulation of organ growth | Gene Ontology | GO:0046622 | *HEY2* | 0.015079757 |
| regulation of epidermis development | Gene Ontology | GO:0045682 | *HEY2* | 0.015079757 |
| cardiac septum morphogenesis | Gene Ontology | GO:0060411 | *HEY2* | 0.015079757 |
| muscle tissue morphogenesis | Gene Ontology | GO:0060415 | *HEY2* | 0.015079757 |
| negative regulation of apoptotic process | Gene Ontology | GO:0043066 | *SNCB, HEY2* | 0.015730734 |
| negative regulation of programmed cell death | Gene Ontology | GO:0043069 | *SNCB, HEY2* | 0.016067827 |
| activating transcription factor binding | Gene Ontology | GO:0033613 | *HEY2* | 0.01614844 |
| anterior/posterior axis specification | Gene Ontology | GO:0009948 | *HEY2* | 0.01614844 |
| cardiac ventricle morphogenesis | Gene Ontology | GO:0003208 | *HEY2* | 0.01614844 |
| myoblast fusion | Gene Ontology | GO:0007520 | *DYRK1B* | 0.01614844 |
| labyrinthine layer development | Gene Ontology | GO:0060711 | *HEY2* | 0.01614844 |
| amino sugar metabolic process | Gene Ontology | GO:0006040 | *GFPT2* | 0.01614844 |
| artery development | Gene Ontology | GO:0060840 | *HEY2* | 0.01614844 |
| negative regulation of DNA binding | Gene Ontology | GO:0043392 | *HEY2* | 0.01614844 |
| regulation of organ growth | Gene Ontology | GO:0046620 | *HEY2* | 0.01614844 |
| muscle organ morphogenesis | Gene Ontology | GO:0048644 | *HEY2* | 0.017216003 |
| syncytium formation | Gene Ontology | GO:0006949 | *DYRK1B* | 0.017216003 |
| syncytium formation by plasma membrane fusion | Gene Ontology | GO:0000768 | *DYRK1B* | 0.017216003 |
| regulation of transcription regulatory region DNA binding | Gene Ontology | GO:2000677 | *HEY2* | 0.017216003 |
| inclusion body | Gene Ontology | GO:0016234 | *SNCB* | 0.017216003 |
| T cell apoptotic process | Gene Ontology | GO:0070231 | *LGALS13* | 0.017216003 |
| cell-cell fusion | Gene Ontology | GO:0140253 | *DYRK1B* | 0.017216003 |
| positive regulation of blood circulation | Gene Ontology | GO:1903524 | *HEY2* | 0.018282448 |
| negative regulation of cell death | Gene Ontology | GO:0060548 | *SNCB, HEY2* | 0.018333999 |
| outflow tract morphogenesis | Gene Ontology | GO:0003151 | *HEY2* | 0.019347775 |
| transcription initiation from RNA polymerase II promoter | Gene Ontology | GO:0006367 | *HEY2* | 0.019347775 |
| smooth muscle cell differentiation | Gene Ontology | GO:0051145 | *HEY2* | 0.020411986 |
| cardiac muscle cell differentiation | Gene Ontology | GO:0055007 | *HEY2* | 0.021475082 |
| histone deacetylase complex | Gene Ontology | GO:0000118 | *HEY2* | 0.021475082 |
| cardiac septum development | Gene Ontology | GO:0003279 | *HEY2* | 0.021475082 |
| transcriptional repressor complex | Gene Ontology | GO:0017053 | *HEY2* | 0.021475082 |
| regulation of lymphocyte apoptotic process | Gene Ontology | GO:0070228 | *LGALS13* | 0.021475082 |
| positive regulation of muscle organ development | Gene Ontology | GO:0048636 | *HEY2* | 0.022537064 |
| positive regulation of muscle tissue development | Gene Ontology | GO:1901863 | *HEY2* | 0.022537064 |
| positive regulation of striated muscle tissue development | Gene Ontology | GO:0045844 | *HEY2* | 0.022537064 |
| columnar/cuboidal epithelial cell differentiation | Gene Ontology | GO:0002065 | *HEY2* | 0.022537064 |
| histone deacetylase binding | Gene Ontology | GO:0042826 | *HEY2* | 0.022537064 |
| axis specification | Gene Ontology | GO:0009798 | *HEY2* | 0.023597934 |
| cardiac ventricle development | Gene Ontology | GO:0003231 | *HEY2* | 0.023597934 |
| vasculogenesis | Gene Ontology | GO:0001570 | *HEY2* | 0.023597934 |
| anatomical structure formation involved in morphogenesis | Gene Ontology | GO:0048646 | *DYRK1B, HEY2* | 0.023842802 |
| embryonic placenta development | Gene Ontology | GO:0001892 | *HEY2* | 0.024657691 |
| cardiac chamber morphogenesis | Gene Ontology | GO:0003206 | *HEY2* | 0.024657691 |
| regulation of heart rate | Gene Ontology | GO:0002027 | *HEY2* | 0.025716338 |
| negative regulation of binding | Gene Ontology | GO:0051100 | *HEY2* | 0.025716338 |
| regulation of leukocyte apoptotic process | Gene Ontology | GO:2000106 | *LGALS13* | 0.025716338 |
| phenol-containing compound metabolic process | Gene Ontology | GO:0018958 | *SNCB* | 0.025716338 |
| regulation of Notch signaling pathway | Gene Ontology | GO:0008593 | *HEY2* | 0.025716338 |
| lymphocyte apoptotic process | Gene Ontology | GO:0070227 | *LGALS13* | 0.025716338 |
| organ growth | Gene Ontology | GO:0035265 | *HEY2* | 0.026773875 |
| epithelial to mesenchymal transition | Gene Ontology | GO:0001837 | *HEY2* | 0.026773875 |
| myotube differentiation | Gene Ontology | GO:0014902 | *DYRK1B* | 0.027830304 |
| regulation of striated muscle tissue development | Gene Ontology | GO:0016202 | *HEY2* | 0.029939842 |
| regulation of muscle organ development | Gene Ontology | GO:0048634 | *HEY2* | 0.029939842 |
| regulation of muscle tissue development | Gene Ontology | GO:1901861 | *HEY2* | 0.029939842 |
| leukocyte apoptotic process | Gene Ontology | GO:0071887 | *LGALS13* | 0.030992952 |
| cardiac chamber development | Gene Ontology | GO:0003205 | *HEY2* | 0.030992952 |
| cardiocyte differentiation | Gene Ontology | GO:0035051 | *HEY2* | 0.030992952 |
| endothelial cell differentiation | Gene Ontology | GO:0045446 | *HEY2* | 0.030992952 |
| epidermal cell differentiation | Gene Ontology | GO:0009913 | *HEY2* | 0.032044959 |
| regulation of epithelial cell differentiation | Gene Ontology | GO:0030856 | *HEY2* | 0.032044959 |
| regulation of DNA binding | Gene Ontology | GO:0051101 | *HEY2* | 0.032044959 |
| negative regulation of neuron apoptotic process | Gene Ontology | GO:0043524 | *SNCB* | 0.032044959 |
| endothelium development | Gene Ontology | GO:0003158 | *HEY2* | 0.033095863 |
| DNA-templated transcription, initiation | Gene Ontology | GO:0006352 | *HEY2* | 0.033095863 |
| muscle cell proliferation | Gene Ontology | GO:0033002 | *HEY2* | 0.033095863 |
| striated muscle cell development | Gene Ontology | GO:0055002 | *HEY2* | 0.034145666 |
| negative regulation of neuron death | Gene Ontology | GO:1901215 | *SNCB* | 0.035194367 |
| phospholipase activity | Gene Ontology | GO:0004620 | *LGALS13* | 0.03624197 |
| muscle cell development | Gene Ontology | GO:0055001 | *HEY2* | 0.03624197 |
| synapse organization | Gene Ontology | GO:0050808 | *SNCB* | 0.03624197 |
| cardiac muscle tissue development | Gene Ontology | GO:0048738 | *HEY2* | 0.037288474 |
| cellular aromatic compound metabolic process | Gene Ontology | GO:0006725 | *SNCB, GFPT2, DYRK1B, HEY2* | 0.037832318 |
| regulation of heart contraction | Gene Ontology | GO:0008016 | *HEY2* | 0.03833388 |
| regulation of neuron apoptotic process | Gene Ontology | GO:0043523 | *SNCB* | 0.03833388 |
| inner ear development | Gene Ontology | GO:0048839 | *HEY2* | 0.039378191 |
| carboxylic ester hydrolase activity | Gene Ontology | GO:0052689 | *LGALS13* | 0.042504558 |
| placenta development | Gene Ontology | GO:0001890 | *HEY2* | 0.042504558 |
| organic cyclic compound metabolic process | Gene Ontology | GO:1901360 | *SNCB, GFPT2, DYRK1B, HEY2* | 0.042793125 |
| positive regulation of developmental growth | Gene Ontology | GO:0048639 | *HEY2* | 0.043544495 |
| regulation of neuron death | Gene Ontology | GO:1901214 | *SNCB* | 0.043544495 |
| neuron apoptotic process | Gene Ontology | GO:0051402 | *SNCB* | 0.044583342 |
| positive regulation of transcription, DNA-templated | Gene Ontology | GO:0045893 | *DYRK1B, HEY2* | 0.045574553 |
| protein-DNA complex assembly | Gene Ontology | GO:0065004 | *HEY2* | 0.045621099 |
| heart contraction | Gene Ontology | GO:0060047 | *HEY2* | 0.045621099 |
| ear development | Gene Ontology | GO:0043583 | *HEY2* | 0.046657768 |
| heart process | Gene Ontology | GO:0003015 | *HEY2* | 0.046657768 |
| protein-DNA complex subunit organization | Gene Ontology | GO:0071824 | *HEY2* | 0.04769335 |
| lipase activity | Gene Ontology | GO:0016298 | *LGALS13* | 0.048727845 |
| Notch signaling pathway | Gene Ontology | GO:0007219 | *HEY2* | 0.048727845 |
| mesenchymal cell differentiation | Gene Ontology | GO:0048762 | *HEY2* | 0.049761256 |
| ammonium ion metabolic process | Gene Ontology | GO:0097164 | *SNCB* | 0.049761256 |
